# Comparative Genomic Analysis Provides Insights into the Evolution and Genetic Diversity of Community-Genotype Sequence Type 72 Staphylococcus aureus Isolates

**DOI:** 10.1128/mSystems.00986-21

**Published:** 2021-09-07

**Authors:** Wangxiao Zhou, Ye Jin, Yanzi Zhou, Yuan Wang, Luying Xiong, Qixia Luo, Yonghong Xiao

**Affiliations:** a State Key Laboratory for Diagnosis and Treatment of Infectious Diseases, National Clinical Research Center for Infectious Diseases, Collaborative Innovation Center for Diagnosis and Treatment of Infectious Diseases, The First Affiliated Hospital, Zhejiang University School of Medicine, Hangzhou, China; Istanbul Medipol University School of Medicine

**Keywords:** *Staphylococcus aureus*, sequence type 72, evolutionary dynamics, transmission, antimicrobial resistance, virulence, mobile genetic elements

## Abstract

Staphylococcus aureus sequence type (ST) 72, the predominant community-associated methicillin-resistant Staphylococcus aureus (CA-MRSA) lineage in South Korea, has emerged as a major cause of bloodstream infection in hospital settings. However, relatively little information is available regarding the genomic characteristics and dissemination of ST72. Here, we characterized the whole-genome sequence of 24 ST72 isolates from China, along with 83 ST72 genomes from global sources. Of these 107 ST72 isolates, 63 were MRSA and 44 were methicillin-susceptible S. aureus (MSSA). Phylogenetic analysis revealed four distinct clades (A, B, C, and D), of which clade D contained only MSSA isolates. By characterizing the evolutionary dynamics of the ST72 lineage, we found that the MRSA from China might not have developed from the MSSA in China. Furthermore, we observed both international transmission of ST72 isolates and interregional transmission within China. The distributions of the SCC*mec* and *spa* types of isolates differed among clades. Additionally, *in silico* analyses revealed that the distributions of resistance genes, virulence genes, and mobile genetic elements (MGEs) also differed among isolates of the four clades. This was especially true for clade D isolates, which had the lowest level of antimicrobial resistance and had obtained specific virulence genes such as *tsst-1* by acquisition of specific MGEs. Notably, ST72 MRSA isolates were more antibiotic resistant than ST72 MSSA isolates, but comparably virulent. Our findings provide insight into the potential transmission and genotypic features of ST72 clones across the globe.

**IMPORTANCE** Understanding the evolution and dissemination of community-genotype ST72 Staphylococcus aureus isolates is important, as isolates of this lineage have rapidly spread into hospital settings and caused serious health issues. In this study, we first carried out genome-wide analysis of 107 global ST72 isolates to characterize the evolution and genetic diversity of the ST72 lineage. We found that the MSSA lineage in China might have evolved independently from the MRSA isolates from China, and that ST72 isolates have the potential to undergo both international transmission and interregional transmission within China. The diversity of isolates correlated with distinct acquisitions of SCC*mec* elements, antibiotic resistance genes, virulence genes, and mobile genetic elements. The comprehensive information on the ST72 lineage emerging from this study will enable improved therapeutic approaches and rapid molecular diagnosis.

## INTRODUCTION

Staphylococcus aureus is an important human pathogen that causes serious hospital- and community-acquired infections worldwide. It asymptomatically colonizes human skin and nasal membranes in about one-third of the human population, and may account for infections with outcomes that range from mild skin lesions to severe diseases, such as necrotizing pneumonia, septicemia, endocarditis, and toxic shock syndrome (1, 2). Until the mid-1990s, methicillin-resistant S. aureus (MRSA) was reported to be solely hospital acquired (HA-MRSA) (3); however, in the late 1990s, it began to be increasingly identified in community-acquired infections (CA-MRSA) (4). Although HA-MRSA tends to infect elderly patients with underlying health issues (5), CA-MRSA tends to infect healthy younger individuals without identified risk factors, potentially causing fatal sepsis and severe pneumonia (6).

Unlike HA-MRSA isolates, CA-MRSA isolates are generally less resistant to antibiotics and often carry the prophage genes *lukS*/*F-PV*, encoding Panton-Valentine leukocidin (PVL) toxin, which might have a role in promoting soft tissue and skin infections (7); however, several CA-MRSA clones are PVL-negative, including the CA-MRSA clone ST72 (8). In addition, CA-MRSA harbors type IV or V staphylococcal cassette chromosome *mec* (SCC*mec*) elements that are smaller than the type I, II, or III SCC*mec* elements carried by HA-MRSA (7, 9). The extraordinary success of CA-MRSA isolates is owing to the combination of two factors, i.e., the low fitness cost of methicillin resistance and elevated virulence (10). Distinct CA-MRSA lineages dominate in different geographical regions, e.g., USA300 in the United States and ST59 in Asia (11, 12).

CA-MRSA ST72 is the most widespread CA-MRSA genotype in South Korea (13). The first report on CA-MRSA in South Korea revealed that ST72 accounted for 35% of all CA-MRSA isolates (14). Since the late 2000s, ST72 MRSA has been introduced into hospitals and has become a major cause of bloodstream infection (BSI) (15). Joo et al. (16) reported that ST72 isolates are tolerant of stressful environments, including desiccation and hypotonic solutions, which could promote their survival in health care facilities. Moreover, although ST72 is relatively susceptible to non-β-lactams, several isolates have undergone rapid evolution of antimicrobial resistance, and some have been identified as vancomycin-intermediate isolates (17, 18). Although isolation of ST72 isolates has been reported sporadically in other countries such as China (19), Japan (18), and Spain (20), no previous study has provided detailed information on the genomic features of the ST72 lineage on a global scale.

We analyzed the genomic features of 24 ST72 strains isolated in China between 2014 and 2019. To characterize the genotypic features and potential transmission of various ST72 isolates on a global scale, 83 additional ST72 isolates with available geographic information were selected for comparison (Table S1 in the supplemental material). We then performed phylogenetic analysis to reconstruct the evolutionary dynamics of the ST72 lineage. We also analyzed the resistance genes, virulence genes, and mobile genetic elements (MGEs) of ST72 isolates. These genomic analyses clarify the genomic differences, evolutionary relationships, and variations in antibiotic resistance and virulence potential in the ST72 lineage.

10.1128/mSystems.00986-21.1 TABLE S1 Information on ST72 isolates analyzed in this study. Download Table S1, DOCX file, 0.04 MB. Copyright © 2021 Zhou et al. 2021 Zhou et al. https://creativecommons.org/licenses/by/4.0/ This content is distributed under the terms of the Creative Commons Attribution 4.0 International license.

## RESULTS

### Geographic distribution of the S. aureus ST72 isolates.

**FIG 1:**
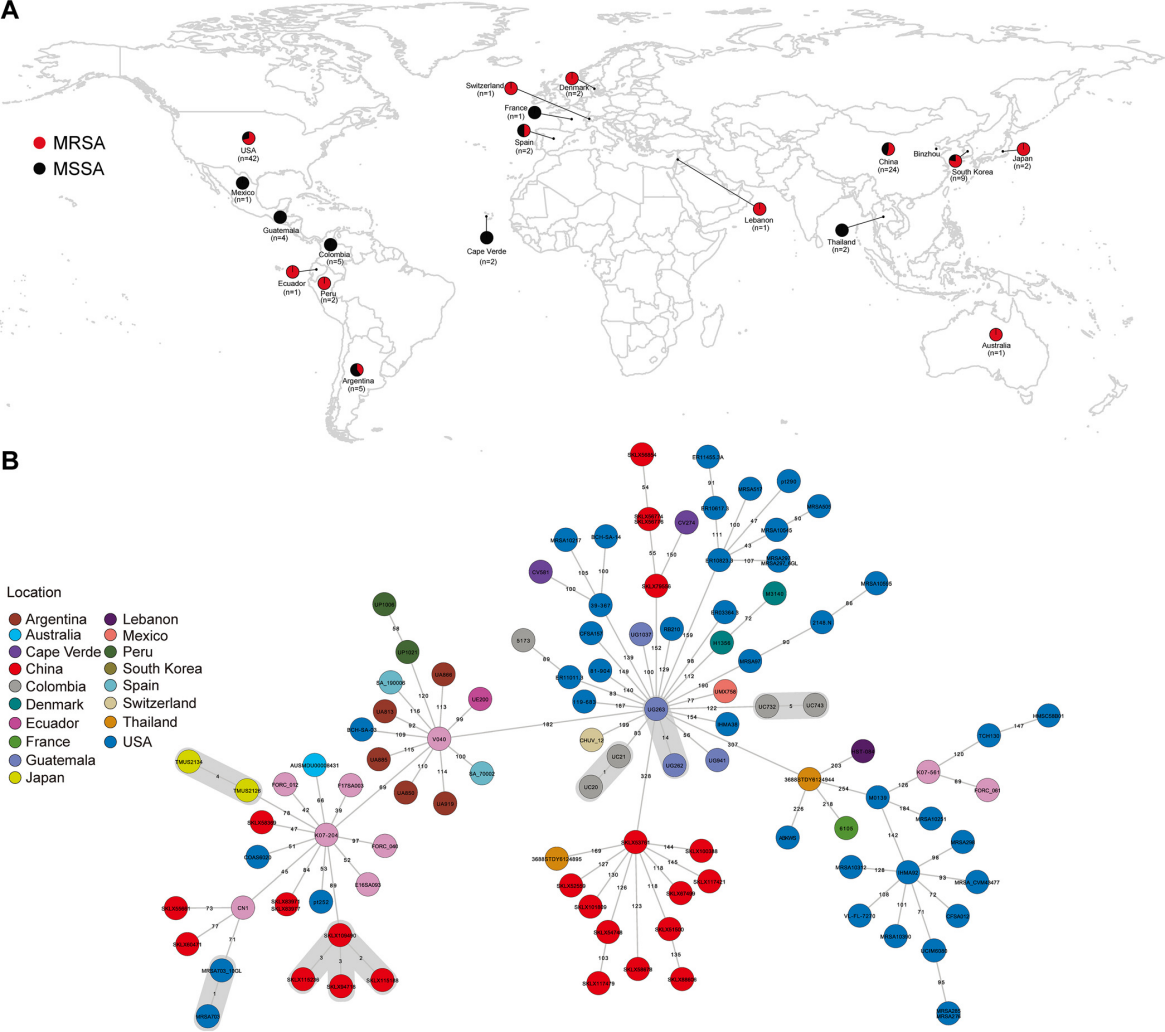
Relationships among ST72 isolates in this study. (A) Geographical distribution of 107 ST72 S. aureus isolates. Red and black dots indicate MRSA and MSSA isolates, respectively. The map was created using R package ggplot2 (https://github.com/tidyverse/ggplot2). (B) MST of ST72 isolates. Each circle represents a ST72 isolate, with colors assigned according to geographic origin. The numbers on the connecting lines illustrate the number of SNPs in a pairwise comparison. The transmission cluster is marked with gray based on the threshold of 23 SNPs.

In this study, we analyzed 107 ST72 S. aureus isolates sampled between 2003 and 2019 (24 from our collection and 83 from the NCBI database), spanning 18 different countries in Asia, Europe, America, Africa, and Oceania (Fig. 1A). The top three countries with the highest numbers of ST72 isolates were the United States (39.25% of the total, *n *= 42), China (22.43%, *n *= 24), and South Korea (8.41%, *n *= 9). Of the 107 ST72 isolates, 63 were methicillin resistant and 44 were methicillin-susceptible S. aureus (MSSA). All isolates from China in our collection (13 MRSA and 11 MSSA) emerged in provinces in close geographic proximity, with the exception of Heilongjiang (Fig. S1A), suggesting potential transmission of ST72 isolates between geographic areas.

10.1128/mSystems.00986-21.3 FIG S1 Relationships among 24 ST72 isolates from China in this study. (A) Geographical distribution of 24 ST72 S. aureus isolates. Red and black dots indicate MRSA and MSSA isolates, respectively. The map was created using R package ggplot2 (https://github.com/tidyverse/ggplot2). (B) Phylogenetic tree of 24 ST72 isolates. Tips are colored by province of isolation. (C) MST of 24 ST72 isolates. Each circle represents a ST72 isolate, with colors assigned according to geographic origin. The numbers on the connecting lines illustrate the number of SNPs in a pairwise comparison. The transmission cluster is marked with gray based on the threshold of 23 SNPs. ND, not determined. Download FIG S1, PDF file, 0.3 MB. Copyright © 2021 Zhou et al. 2021 Zhou et al. https://creativecommons.org/licenses/by/4.0/ This content is distributed under the terms of the Creative Commons Attribution 4.0 International license.

### Phylogenomic analysis of the ST72 lineage.

**FIG 2:**
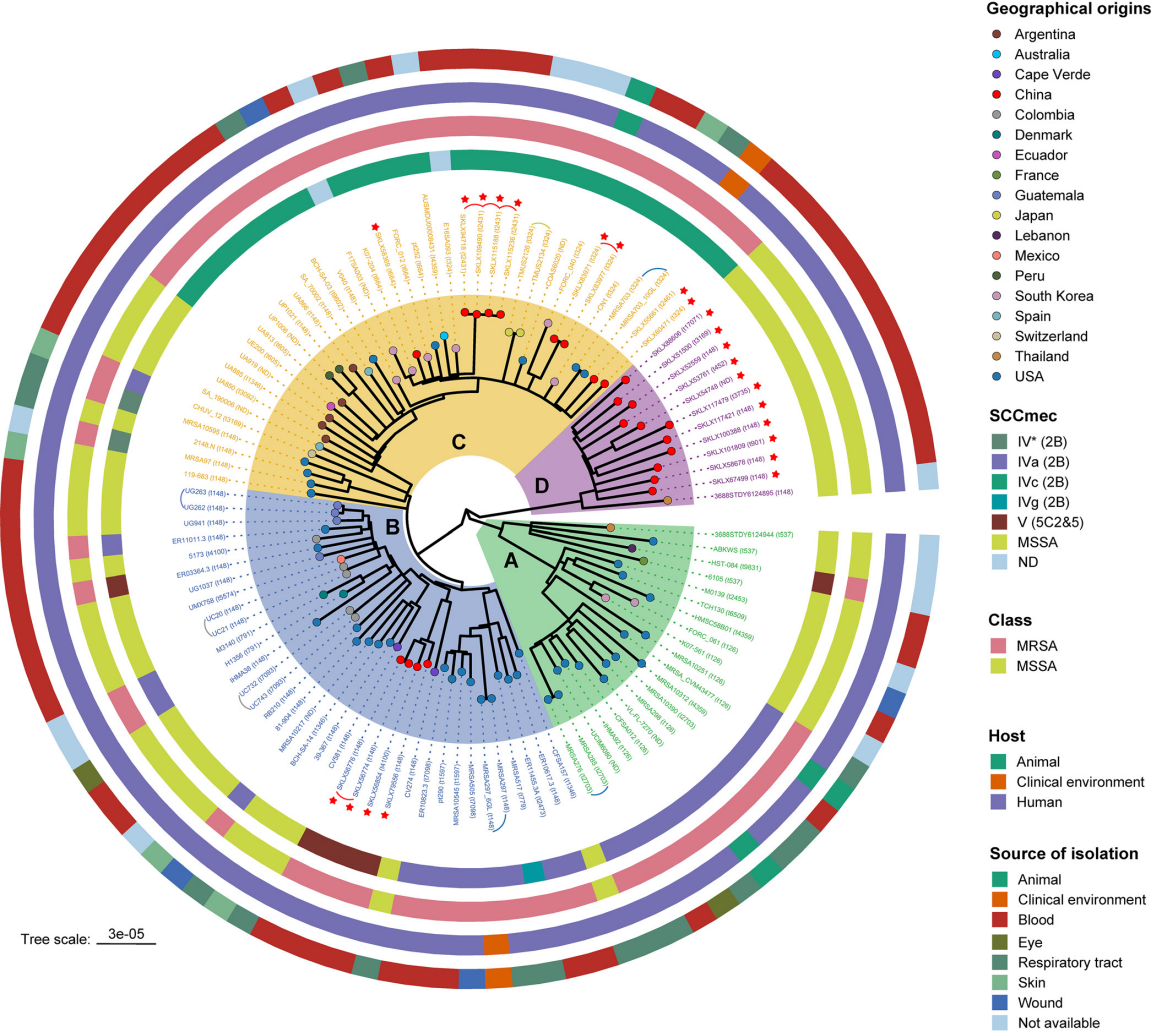
Phylogenetic structure of 107 ST72 isolates. Clades A, B, C, and D are marked by background color fill. Tips are colored according to country of isolation. Data on the SCC*mec* type, MRSA/MSSA classification, host type, and source of isolation are mapped on the tree (from inner to outer circle). Red asterisks indicate the isolates obtained from our collection. Curved lines indicate isolates belonging to same transmission. The *spa* types of each isolate are shown in parentheses near the isolate name. ND, not determined.

To analyze the evolutionary position and genetic diversity of the ST72 isolates examined in this study, we determined single nucleotide polymorphisms (SNPs) within the core genome among all 107 ST72 isolates. Phylogenomic analysis based on 6,345 core SNPs revealed a diverse population structure containing four distinct clades, A through D. The paired SNP distances of isolates between four clades varied from 182 to 488 (median, 368; average, 336), suggesting that the isolates from different clades were distantly related (Fig. 1B). As shown in Fig. 2, clades A, B, and C contained both MRSA and MSSA isolates, whereas clade D contained only MSSA isolates from China (*n *= 11) and Thailand (*n *= 1). In addition, the isolates collected from China and the United States were distributed across three clades of the tree, indicating that isolates from these two countries had greater variability in the core genomic regions (Fig. 2).

Notably, when we mapped the geographic origins of the ST72 lineage onto the phylogenetic tree, we found no evidence for an association between neighboring branches and their locations, regardless of clade (except clade D). By contrast, some isolates clustered with others isolated from different countries, with paired SNP distances ranging from 47 to 488 (median, 265; average, 288). In particular, the four ST72 MRSA isolates collected from the Affiliated Hospital of Binzhou Medical College in 2019 (Binzhou, China) clustered closely with the geographically close isolate E16SA093 from South Korea (isolated in 2016) from clade C (Fig. 2), with paired SNP distance varying from 95 to 98. In addition, using 23 SNPs as the maximum pairwise distance of isolates from the same transmission (21), we identified 10 independent small-scale transmissions across the tree (except clade D) that occurred in the United States, China, Japan, Switzerland, and Guatemala (Fig. 1B and Fig. 2). Of these, two transmission events occurred between patients and the clinical environment in two U.S. hospitals (22).

We also carried out a phylogenetic analysis of 24 ST72 isolates from China. The results revealed no significant boundary existed between isolates from different provinces of China. For instance, isolate SKLX60417, collected from Jiangsu province, was closely related to isolate SKLX55661, collected from Zhejiang province (Fig. S1C). Of note, the paired SNP distances of isolates between these provinces varied from 87 to 452 (median, 365; average, 301) (Fig. S1B).

### Time scale of the emergence of global ST72 isolates.

**FIG 3:**
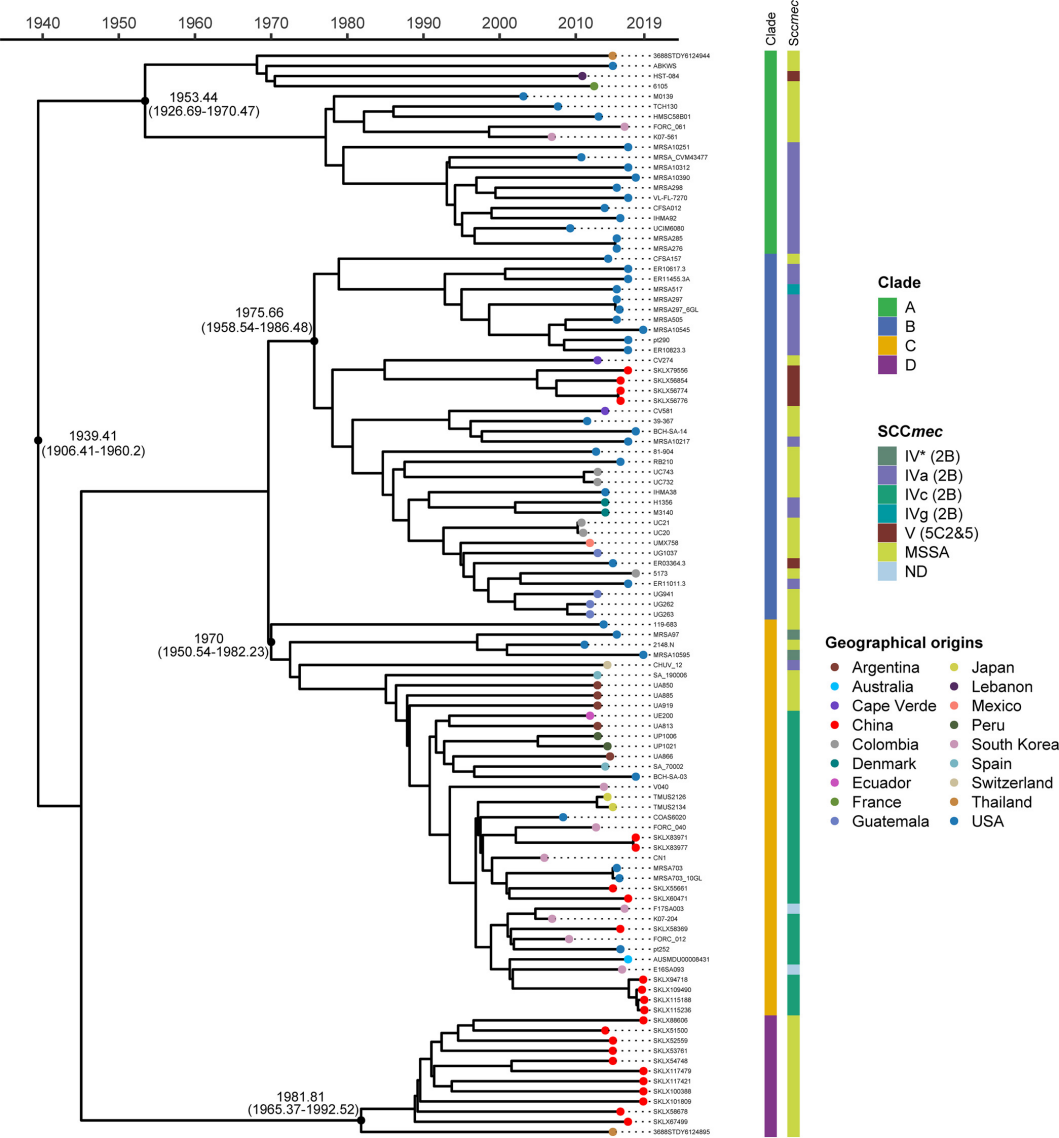
Time-based tree of the 107 ST72 isolates. Characteristics of isolates are shown on the right, including clades, SCC*mec* type, and geographical origin (mapped on the tips). Selected divergence time and 95% HPD intervals are shown at the nodes.

To further assess the time origin of the ST72 lineage, we used a Bayesian approach to explore the temporal spread of the ST72 isolates. The results revealed that the median time of divergence of the ST72 lineage from its most recent common ancestor (MRCA) was around 1939.41 (95% highest posterior density [HPD] interval, 1906.41 to 1960.2). However, the divergence of the four clades in the ST72 lineage occurred more recently; the MRCA of clade A isolates probably emerged in 1953.44, clade B in 1975.66, clade C in 1970, and clade D in 1981.81 (Fig. 3). This finding indicates that the expansion of ST72 occurred at least several decades after its emergence. In addition, the core genome of the ST72 isolates has accumulated variation at a rate of 1.3 × 10^−6^ substitutions per nucleotide site per year (95% HPD interval, 8.87 × 10^−7^ to 1.72 × 10^−6^), similar to the rate of other S. aureus lineages (23, 24).

### SCC*mec* acquisition and *spa* typing of ST72 isolates.

SCC*mec* typing identified only two SCC*mec* types (types IV [2B] and V [5C2&5]) among 63 ST72 MRSA isolates. The most common was type IV (2B), which comprised 55 isolates (90.16%, 55/61), whereas type V (5C2&5) comprised 6 isolates (9.84%, 6/61) (Fig. 2). SCC*mec* type IV (2B) could be further divided into three subtypes: IVa (2B), IVc (2B), and IVg (2B). This finding indicates acquisition of different SCC*mec* elements during the development of methicillin resistance in the ST72 MSSA lineage. Notably, most clade A MRSA isolates harbored SCC*mec* type IVa (2B) (91.67%, 11/12), and only one carried type V (5C2&5); clade B MRSA isolates carried three SCC*mec* types, including type IVa (2B) (66.67%, 12/18), IVg (2B) (5.56%, 1/18), and V (5C2&5) (27.28%, 5/18). Type IVc (2B) was only detected in the majority of clade C MRSA isolates (84.85%, 28/33), and none of the isolates within this clade carried type V (5C2&5) (Fig. 2).

The diversity of *spa* types within all 107 ST72 isolates was confirmed by genotypic *spa* typing. Twenty-nine *spa* types were identified, of which the top three types were *spa* t148 (31.78%, 34/107), t324 (9.35%, 10/107), and t126 (6.54%, 7/107). In addition, we noted that isolates within different clades exhibited considerable diversity of *spa* types (Fig. 2). Notably, in this regard, the *spa* types assigned to isolates of clade D were similar to each other and all belonged to a single *spa* clonal complex (*spa*-CC324), suggesting that this *spa* type (*spa*-CC324) is characteristic of the ST72 clade D MSSA lineage.

### Distributions of AMR genes in the ST72 lineage.

**FIG 4:**
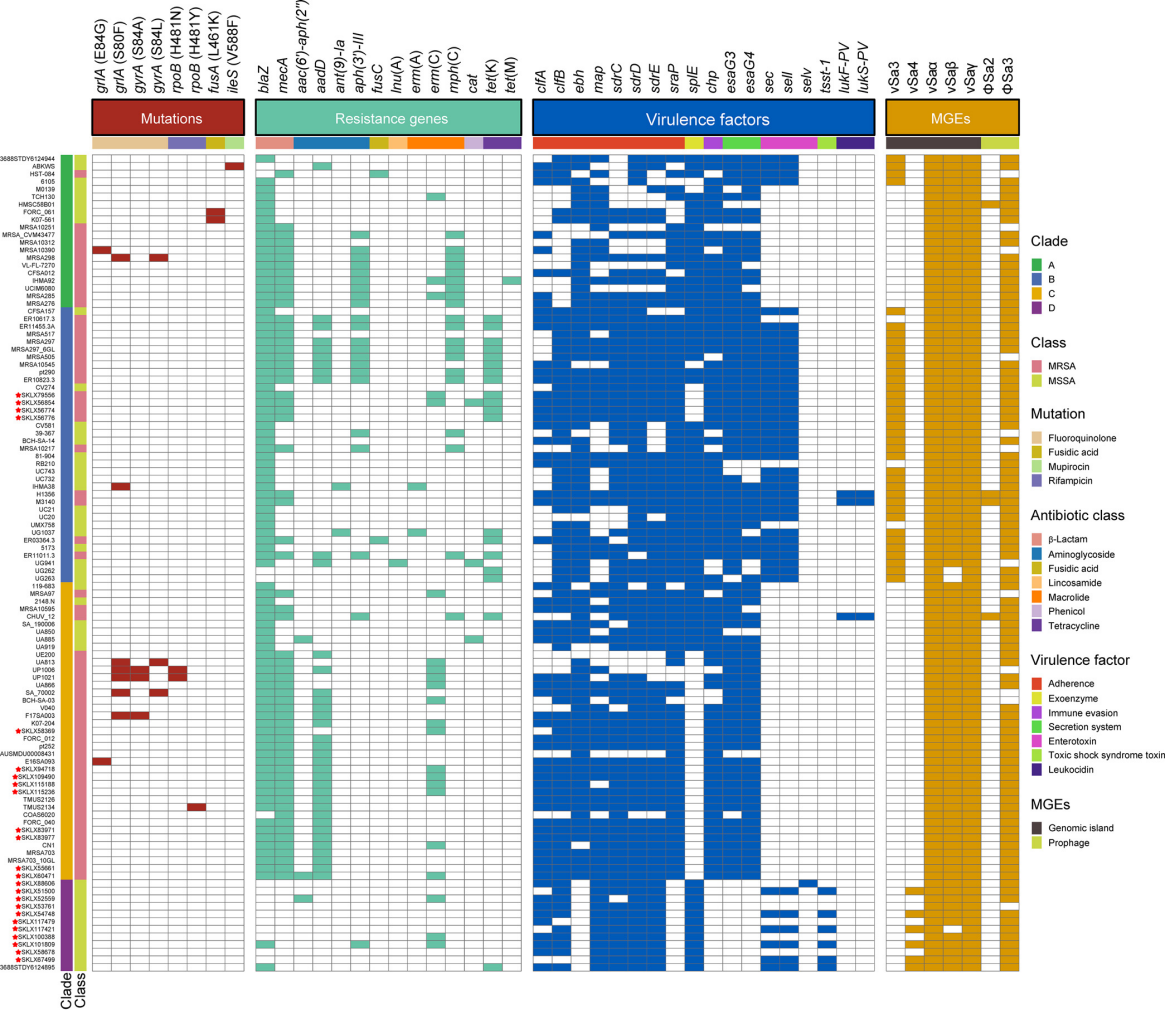
Distributions of mutations, AMR genes, non-core-virulence genes, and MGEs in the ST72 lineage. Squares, which are colored by trait category, represent the presence of the trait examined. Red asterisks indicate the isolates obtained from our collection.

**FIG 5:**
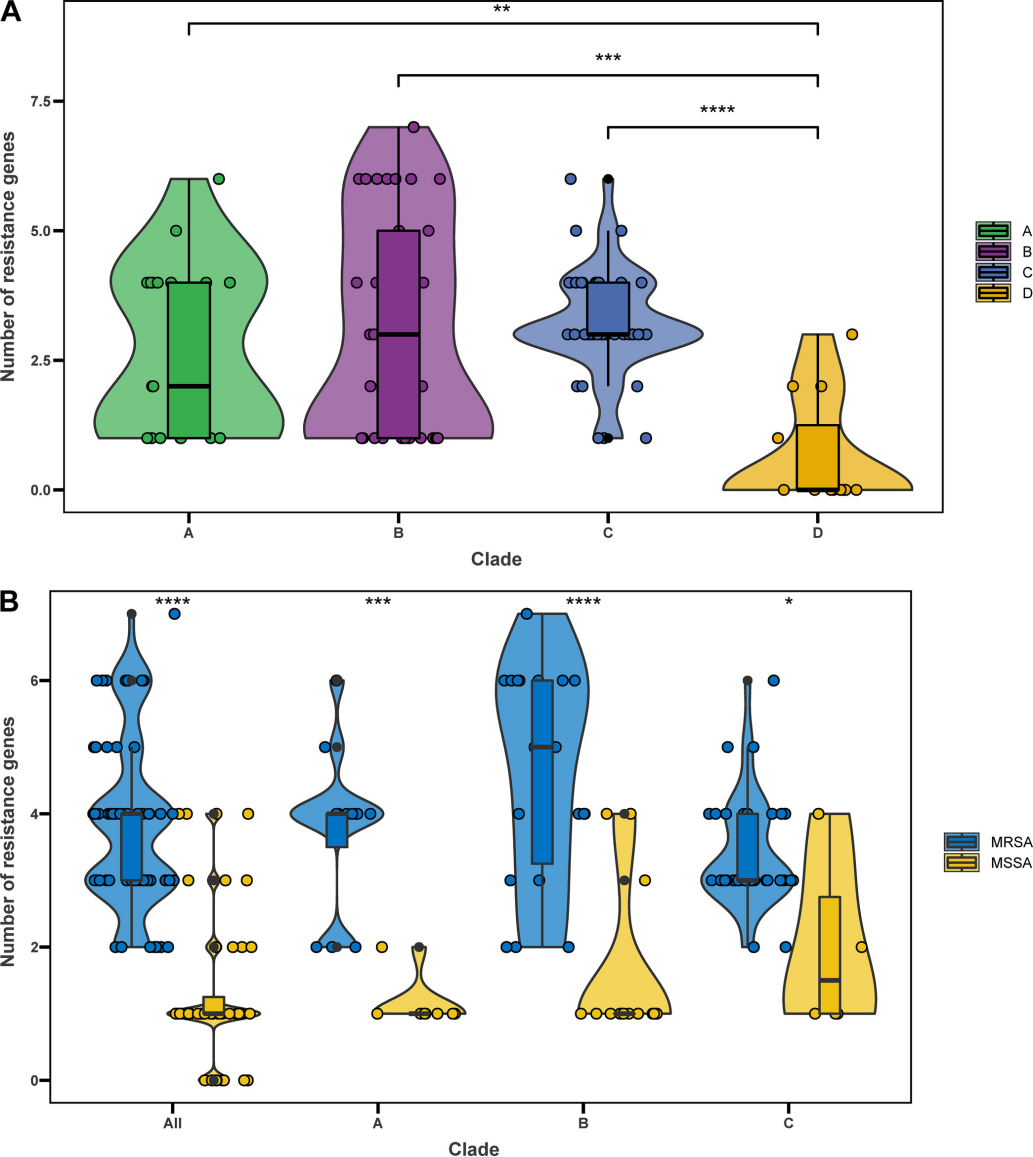
AMR genes in ST72 isolates. (A) Number of acquired AMR genes per isolate in different clades. (B) Number of acquired AMR genes per isolate in MRSA and MSSA isolates from different clades. *, *P < *0.05; **, *P < *0.01; ***, *P < *0.001; ****, *P < *0.0001.

Given the clinical importance of antimicrobial resistance (AMR) in S. aureus, we compared the distributions of AMR genes in ST72 isolates from different clades. A total of 14 AMR genes associated with seven classes of antimicrobial agents (β-lactam, aminoglycoside, fusidic acid, lincosamide, macrolide, phenicol, and tetracycline) were present in isolates from the four clades; 45% (9/20, clade A), 47.2% (17/36, clade B), 28.2% (11/39, clade C), and 8.33% (1/12, clade D) of isolates from the corresponding clades were multidrug resistant (Fig. 4). Moreover, isolates from clades A, B, and C had significantly more AMR genes than those from clade D (P < 0.01) (Fig. 5A). As shown in Fig. 4, the distributions of some AMR genes differed among isolates within the four clades, indicating that isolates of different clades tended to acquire specific antibiotic resistance genes. Notably, detection of gene mutations corresponding to AMR revealed a low frequency of such events in all clades of the ST72 lineage (Fig. 4).

In each clade, MRSA isolates had more AMR genes than MSSA isolates (*P < *0.05) (Fig. 5B). Furthermore, *blaZ*, *aadD*, *aph*(*3′*)*-III*, *erm*(C), and *mph*(C) were detected in more MRSA than MSSA isolates (P < 0.05) (Table 1), indicating that MRSA isolates exhibit a higher degree of AMR than MSSA isolates.

**TABLE 1:** Distribution of antibiotic resistance and virulence genes between ST72 MRSA and ST72 MSSA isolates

Category	Gene	No. (%) of isolates		
		MRSA (n = 63)	MSSA (n = 44)	*P* value
Antibiotic resistance	*blaZ*	61 (96.83)	31 (70.45%)	<0.001
	*mecA*	63 (100)	0 (0)	<0.001
	*aadD*	35 (55.56)	2 (4.55)	<0.001
	*aph(3′)-III*	20 (31.75)	2 (4.55)	0.001
	*erm*(C)	18 (28.57)	4 (9.09)	0.04
	*mph*(C)	19 (30.16)	1 (2.27)	<0.001
Virulence	*ebh*	57 (90.48)	28 (63.64)	0.03
	*sraP*	59 (93.65)	25 (56.82)	<0.001
	*splE*	28 (44.44)	41 (93.18)	<0.001
	*esaG3*	60 (95.24)	26 (59.09)	<0.001
	*esaG4*	58 (92.06)	24 (54.55)	<0.001

### Antibiotic susceptibilities of the ST72 lineage from China.

We also profiled the *in vitro* susceptibility of the 24 ST72 isolates from China to 16 common used antibiotics (Table 2). The presence of drug-resistance genes in these isolates was consistent with their antimicrobial susceptibility profiles, highlighting genome sequencing as a potential tool to assist clinicians with the treatment of complicated S. aureus infections.

**TABLE 2:** Antimicrobial susceptibility of 24 ST72 isolates from China^*a*^

Strain	ERY	CLI	OXA	PEN	SXT	TCY	VAN	RIF	CIP	LVX	MFX	GEN	AMK	TGC	LNZ	DAP
SKLX58369	R	R	R	R	S	S	S	S	S	S	S	S	S	S	S	S
SKLX83971	S	S	R	R	S	S	S	S	S	S	S	S	R	S	S	S
SKLX83977	S	S	R	R	S	S	S	S	S	S	S	S	R	S	S	S
SKLX60471	R	R	R	R	S	S	S	S	S	S	S	S	R	S	S	S
SKLX94718	R	R	R	R	S	S	S	S	S	S	S	S	R	S	S	S
SKLX109490	R	R	R	R	S	S	S	S	S	S	S	S	R	S	S	S
SKLX115188	R	R	R	R	S	S	S	S	S	S	S	S	R	S	S	S
SKLX115236	R	R	R	R	S	S	S	S	S	S	S	S	R	S	S	S
SKLX55661	S	S	R	R	S	S	S	S	S	S	S	S	R	S	S	S
SKLX56774	S	S	R	R	S	R	S	S	S	S	S	S	S	S	S	S
SKLX56776	S	S	R	R	S	R	S	S	S	S	S	S	S	S	S	S
SKLX56854	R	R	R	R	S	R	S	S	S	S	S	S	S	S	S	S
SKLX79556	R	R	R	R	S	R	S	S	S	S	S	S	S	S	S	S
SKLX101809	R	R	S	R	S	S	S	S	S	S	S	S	R	S	S	S
SKLX58678	S	S	S	S	S	S	S	S	S	S	S	S	S	S	S	S
SKLX67499	S	S	S	S	S	S	S	S	S	S	S	S	S	S	S	S
SKLX88606	S	S	S	S	S	S	S	S	S	S	S	S	S	S	S	S
SKLX100388	R	R	S	S	S	S	S	S	S	S	S	S	S	S	S	S
SKLX117421	S	S	S	S	S	S	S	S	S	S	S	S	S	S	S	S
SKLX117479	S	S	S	S	S	S	S	S	S	S	S	S	S	S	S	S
SKLX54748	S	S	S	S	S	S	S	S	S	S	S	S	S	S	S	S
SKLX52559	R	R	S	S	S	S	S	S	S	S	S	S	S	S	S	S
SKLX51500	S	S	S	S	S	S	S	S	S	S	S	S	S	S	S	S
SKLX53761	S	S	S	S	S	S	S	S	S	S	S	S	S	S	S	S

a R, resistant; S, susceptible; ERY, erythromycin; CLI, clindamycin; OXA, oxacillin; PEN, penicillin G; SXT, trimethoprim-sulfamethoxazole; TCY, tetracycline; VAN, vancomycin; RIF, rifampin; CIP, ciprofloxacin; LVX, levofloxacin; MFX, moxifloxacin; GEN, gentamicin; AMK, amikacin; TGC, tigecycline; LNZ, linezolid; DAP, daptomycin.

### Presence of virulence genes in the ST72 lineage.

**FIG 6:**
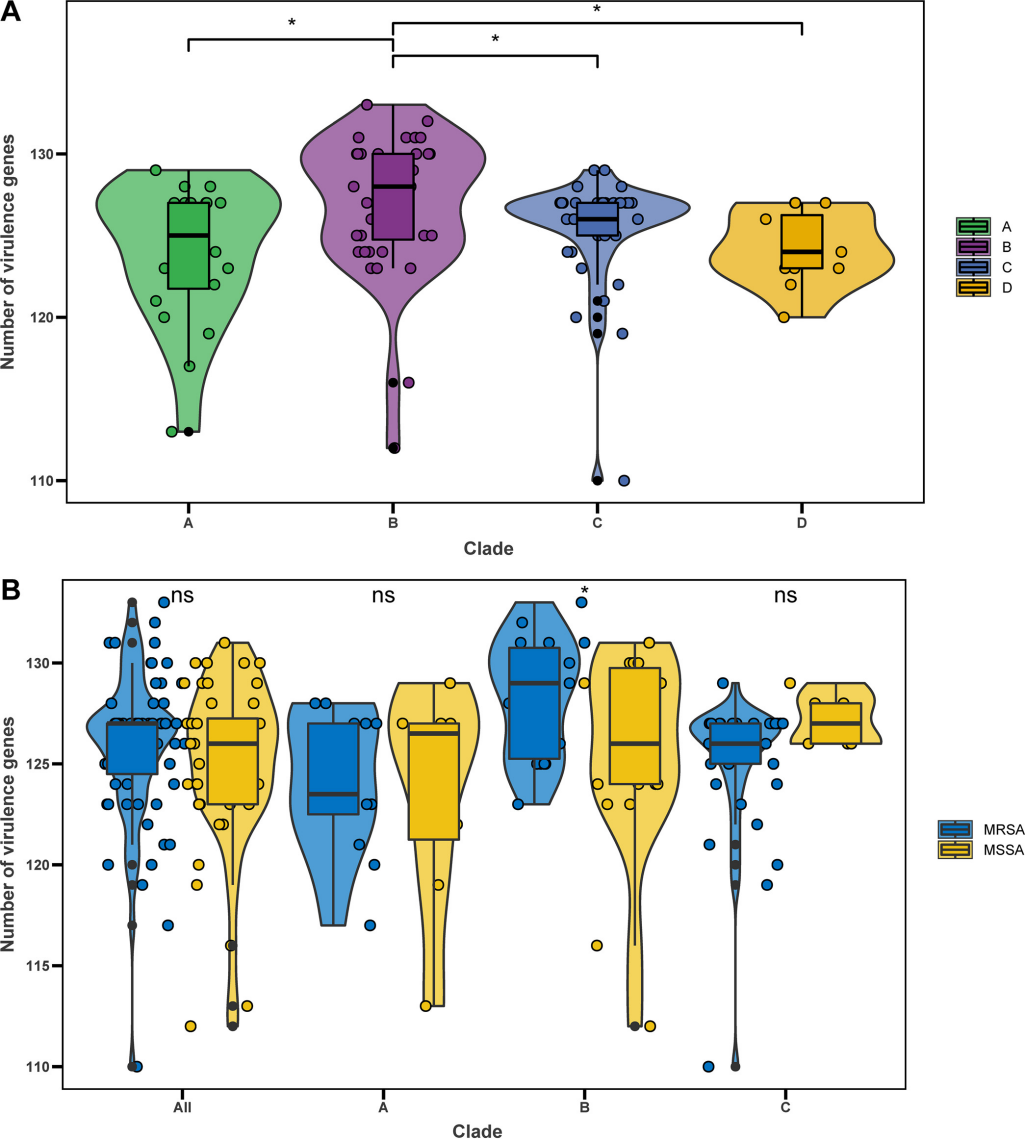
Virulence genes in ST72 isolates. (A) Number of virulence genes per isolate in different clades. (B) Number of virulence genes per isolate in MRSA and MSSA isolates from different clades. *, *P < *0.05; ns, no significant difference between groups.

We next performed a targeted analysis of all known virulence genes in S. aureus to assess the pathogenic potential of isolates within the four clades. We identified a total of 120 virulence genes from 107 ST72 isolates. The number of virulence genes was significantly higher in isolates of clade B than in isolates of any other clade (all *P < *0.05) (Fig. 6A). A series of genes associated with adhesion, exoenzyme, immune evasion, the type VII secretion system, hemolysin (including *hla*), iron uptake, phenol-soluble modulins (PSMs), exotoxin (*set*), exfoliative toxin (*eta*), leukocidin (*lukD* and *lukE*), and enterotoxin (*seg*, *sei*, *selm*, *seln*, *selo*, and *selu2*) were widely distributed among isolates of each clade (≥85% isolates per clade) (Table S2), suggesting that these genes are the core virulence genes of the ST72 lineage. Moreover, these core virulence genes accounted for about 85% (102/120) of all virulence genes detected in ST72 isolates.

10.1128/mSystems.00986-21.2 TABLE S2 Distribution of core virulence genes in the ST72 lineage (≥85% isolates per clade). Download Table S2, DOCX file, 0.02 MB. Copyright © 2021 Zhou et al. 2021 Zhou et al. https://creativecommons.org/licenses/by/4.0/ This content is distributed under the terms of the Creative Commons Attribution 4.0 International license.

Furthermore, with the exception of core virulence genes, the distributions of some other virulence genes differed among isolates of each clade (Fig. 4). In particular, the genes encoding Panton-Valentine leukocidins (*lukS-PV* and *lukF-PV*) were only identified in three isolates of clade B and clade C, whereas the enterotoxin gene *selv* and toxic shock syndrome toxin gene *tsst-1* were only detected in clade D isolates.

The number of virulence genes did not significantly differ between MRSA and MSSA isolates (median 127 versus 126, *P > *0.05) (Fig. 6B). Further, except for *ebh*, *sraP*, *splE*, *esaG3*, and *esaG4* (*P < *0.05) (Table 2), the abundance of other virulence genes did not significantly differ between MRSA and MSSA isolates. Notably, however, for isolates within clade B, MRSA isolates contained significantly more virulence genes than MSSA isolates (median 129 versus 126, *P < *0.05) (Fig. 6B).

In contrast to the epidemic CA-MRSA USA300 (ST8) from the United States and ST59 from Asia, all ST72 isolates lacked virulence genes associated with exoenzyme (*vWbp*, *coa*, and *splE*), immune evasion (*cap5ACEFGIJKNO*, *cap8HIJK*, and *capFGLMP*), and enterotoxin (*selq*, *selk* and *seb*) (Fig. S2). The absence of these genes might decrease the invasion potential of ST72 relative to USA300 and ST59.

10.1128/mSystems.00986-21.4 FIG S2 Network diagram of all virulence genes (VGs) identified in sequence types ST72, ST8 (USA300), and ST59 (M013). “Shared” indicates VGs are present in all STs, “Accessory” indicates VGs are present in two STs, and “ST-specific” indicates VGs are present in a specific ST. Download FIG S2, PDF file, 0.7 MB. Copyright © 2021 Zhou et al. 2021 Zhou et al. https://creativecommons.org/licenses/by/4.0/ This content is distributed under the terms of the Creative Commons Attribution 4.0 International license.

### Pathogenicity islands and prophages in ST72 isolates.

**FIG 7:**
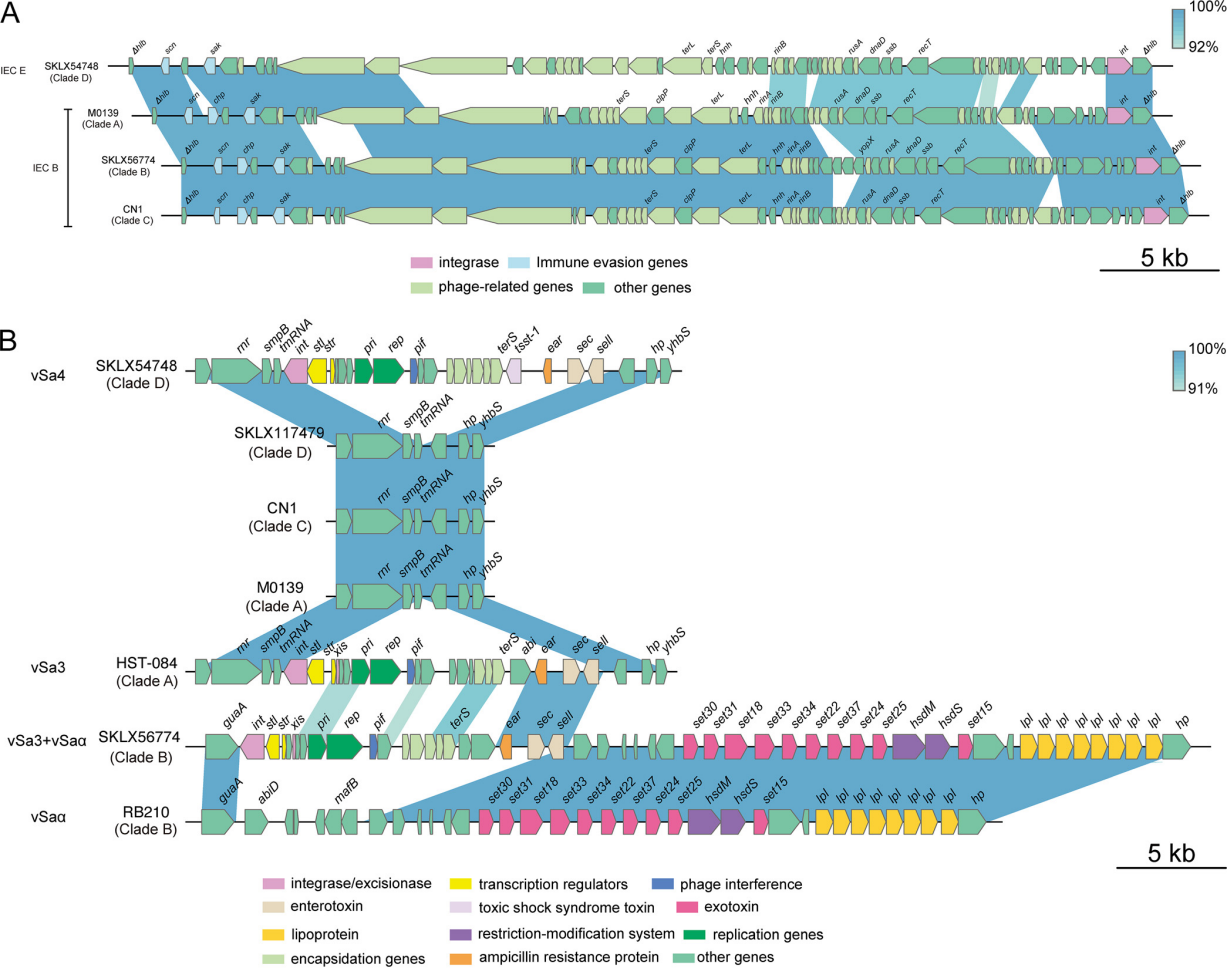
Comparison of ΦSa3 and pathogenicity islands between ST72 isolates in each clade. (A) Comparison of ΦSa3 between ST72 isolates in each clade. (B) Comparison of pathogenicity islands between ST72 isolates in each clade. Genes are represented by arrowed boxes and colored based on gene function classification.

We next examined the distribution of pathogenicity islands and prophages in ST72 isolates because they are significant components of the S. aureus genome that might contribute to the evolution of virulence. The results revealed that most of the isolates from each clade (>85%) harbored three pathogenicity islands, vSaα, vSaβ, and vSaγ, as well as one prophage, ΦSa3 (Fig. 4). However, ΦSa3 of clade D isolates harbored immune evasion cluster (IEC) type E (IEC E, encoding *scn*-*sak*), whereas ΦSa3 of isolates within all other clades carried IEC B (encoding *scn*-*chp*-*sak*) (Fig. 7A). The vSa3 pathogenicity island, which encodes enterotoxin genes *sec* and *sell*, was mainly detected in clade B isolates (91.67%, 33/36) and a few clade A isolates (20%, 4/20); however, between the two clades, vSa3 shared a small number of homologous genes, and vSa3 carried by clade B isolates was integrated into vSaα by recombination, forming a mosaic structure. This finding implies that the vSa3 pathogenicity islands in clade A and clade B isolates are unlikely to have evolved from a common ancestor (Fig. 7B). The vSa4 pathogenicity island, encoding *sec*, *sell*, and *tsst-1*, was only found in half of the clade D isolates (6/12).

In all isolates of clade D, we identified an unknown prophage of 37,817 bp that was integrated immediately downstream of the glutamine synthetase gene *glnA* and flanked by a pair of 57-bp perfect direct repeats (Fig. S3A). The prophage contained 48 open reading frames (ORFs) arranged in a modular fashion; the genome consisted of genes involved in lysis, lysogeny, DNA replication and metabolism, capsid formation and assembly, and head and tail morphogenesis. A BLAST search against the NCBI nucleotide database indicated that this prophage resembled those found in nine S. aureus chromosomes (>98% identity and ≥80% coverage). However, even the two prophages (harbored by S. aureus NRS152 and S. aureus XQ, respectively) that shared the highest sequence similarities with this unknown prophage, only covered 87% of its entire genome. The phylogenetic relationship indicated a single ancestral acquisition of the unknown prophage in clade D isolates (Fig. S3B).

10.1128/mSystems.00986-21.5 FIG S3 Comparison of the unknown prophage with similar prophages. (A) Circular representation of the unknown prophage and comparative genomic analysis with other similar prophages. Circle 1 (from outer to inner circle) shows the genes encoded on the unknown prophage; the colors of genes are assigned based on gene function classification. Circles 2 to 10 refer to homologous regions of S. aureus 93b_S9 (CP010952.1), 27 (CP022717.1), 78 (CP022682.1), UP_426 (CP047797.1), UP_551 (CP047794.1), 128 (CP022897.1), 422 (CP022898.1), XQ (CP013137.1), and NRS153 (CP026067.1) relative to the unknown prophage. Circles 11 and 12 represent GC content and GC skew of the unknown prophage, respectively. Direct repeats are indicated by arrows. (B) Phylogenetic analysis of the unknown prophage with other similar prophages. Download FIG S3, PDF file, 1.9 MB. Copyright © 2021 Zhou et al. 2021 Zhou et al. https://creativecommons.org/licenses/by/4.0/ This content is distributed under the terms of the Creative Commons Attribution 4.0 International license.

### Genetic changes differentiating isolates of each clade.

To obtain insights into the SNP-based evolutionary analysis of the ST72 population, we searched for mutations that could differentiate the ST72 isolates of distinct clades. We identified a total of 40, 4, and 98 SNPs associated with clades A, B, and D, respectively, but no clade-specific SNPs within clade C isolates (Table S3). Strikingly, five clade A-specific nonsynonymous SNPs were located in genes involved in bacterial adhesion (*atl*, *clfA*, *fnbA*, and *icaB*) and capsule (*capD*), and seven clade D-specific nonsynonymous SNPs were related to bacterial adhesion (*sasH*), aurolysin (*aur*), capsule (*capD* and *capI*), exotoxin (*set*), and iron uptake (*isdA* and *sfaD*).

10.1128/mSystems.00986-21.2 TABLE S3 Clade-specific SNPs as identified by Scoary. Download TABLE S3 DOCX file, 0.03 MB. Copyright © 2021 Zhou et al. 2021 Zhou et al. https://creativecommons.org/licenses/by/4.0/ This content is distributed under the terms of the Creative Commons Attribution 4.0 International license.

## DISCUSSION

The community-genotype S. aureus ST72 lineage, which is predominant in South Korea (13), has emerged and gradually increased in hospital settings, similar to other hospital-genotype isolates, such as ST5 and ST239 in Asia. Although ST72 isolates have rarely been reported in other countries (18–20), the 107 ST72 isolates analyzed in this study covered 18 countries on five different continents and were isolated between 2003 and 2019. The most frequently observed isolates were from the United States (*n *= 42), where isolation of ST72 isolates has not been previously reported, followed by China (*n *= 24), which has reported only sporadic isolation of ST72 isolates (19). Notably, ST72 is the trilocus variant of ST8, while ST8 is the most epidemic clone in the United States. The results of biogeography analysis indicated that the United States may be the more probable ancestral origin of ST72 isolates from which these clones began to diversify (Fig. S4), but this could also be the result of sampling bias. These findings suggest that the potential dissemination of ST72 clones around the world has been underestimated, and that large-scale monitoring will be necessary.

10.1128/mSystems.00986-21.6 FIG S4 Phylogeographic analysis of global ST72 isolates based on the Bayesian binary MCMC method in RASP v4.2. Download FIG S4, PDF file, 0.4 MB. Copyright © 2021 Zhou et al. 2021 Zhou et al. https://creativecommons.org/licenses/by/4.0/ This content is distributed under the terms of the Creative Commons Attribution 4.0 International license.

A recent study by Jian et al. (25) reconstructed the phylogeny of ST5 strains isolated from an affiliated tertiary hospital in China between 2008 and 2018. Those authors reported that ST5 MSSA isolates evolved independently in a manner distinct from ST5 MRSA isolates. Similarly, using phylogenetic reconstruction in conjunction with temporal information of the ST72 isolates, we found that all MSSA isolates from China formed a separate clade (clade D) in the tree with an MSSA isolate from Thailand, suggesting the ST72 MRSA and MSSA isolates of China might have evolved independently, i.e., the ST72 MRSA clones in China did not evolve from the susceptible isolates. However, our phylogenetic analysis also revealed that in some other countries, including the United States, several human-adapted ST72 MRSA isolates were much more closely related to human-adapted MSSA isolates, suggesting they may have evolved from the latter by acquisition of SCC*mec* elements (Fig. 2).

In addition, we identified potential international transmission of ST72 isolates, as some isolates from different countries clustered closely with each other. However, the paired SNP distances of isolates between countries surpassed the 23 SNPs suggested by Uhlemann et al. (21) as the maximum pairwise distance of isolates from direct transmission, implying that the epidemic links between clustered isolates from different countries may not be recent and direct; rather, these isolates might have undergone local expansion. Notably, our analysis of ancestral area reconstruction revealed that ST72-SCC*mec* IVc (2B) isolates from China in this study might have originated from South Korea (Fig. S4); in particular, four ST72-SCC*mec* IVc (2B) MRSA isolates from the Affiliated Hospital of Binzhou Medical College (Binzhou, China) were most closely genetically related to isolate E16SA093 from South Korea (Fig. 2, Fig. 3, and Fig. S4). Given the relatively close relationship between isolates from Binzhou and South Korea (paired SNP distance: 95 to 98) and the small geographical distance between the two locations (Fig. 1A), we speculate that international transmission of ST72 isolates might have occurred between South Korea and China. Similarly, our analysis of ST72 isolates from China showed that although the paired SNP distances of isolates from different provinces varied from 87 to 452 (Fig. S1B), the possibility of interregional dissemination in China remained due to the small geographical distances among these provinces (with the exception of Heilongjiang province).

In contrast to HA S. aureus, CA S. aureus has been categorized as susceptible to non-β-lactam antibiotics (26). However, among all isolates of the ST72 lineage analyzed in this study, we identified 14 AMR genes classified into seven antimicrobial classes. Nearly half of the isolates of clades A (45%) and B (47.2%) exhibited multidrug resistance (Fig. 3); hence, we speculate that isolates within these two clades are more likely to spread and thrive in hospitals or originate from hospital settings. Overall, we detected significantly more antibiotic-resistance genes among MRSA than MSSA isolates (Fig. 5B); this could explain why clade D isolates, which were all MSSA isolates, had a low rate of genotypic multidrug resistance. Strikingly, unlike other pandemic S. aureus clones, such as ST22 EMRSA-15, the success of which could be partially attributed to mutations within resistance genes (27), we identified a low mutation frequency in resistance genes carried by isolates of any clade of the ST72 lineage (Fig. 3). This finding indicates that gene mutations play only a limited role during the development of resistance in the ST72 lineage.

Virulence is an important determinant of whether isolates can induce clinical infections. Of the 120 virulence genes identified from all isolates, 102 were members of the core virulence genes, implying they might play a role in host adaption and evolution of virulence in the ST72 lineage. CA-ST72 isolates generally do not have PVL-encoding genes (16). Although three ST72 isolates in the current study were PVL positive and harbored PVL-encoding genes in ΦSa2 PVL prophages (2 intact), they were located within separate clades (clade B and clade C) (Fig. 2), indicating independent acquisition of PVL-encoding genes. Interestingly, the distribution of some virulence genes in clade D isolates was associated with acquisition of specific MGEs. In contrast to isolates of the other clades, clade D isolates exclusively harbored IEC E lacking *chp* in the ΦSa3 prophage, the vSa4 pathogenicity island containing *tsst-1*, and a novel 38-kb prophage encoding genes of unknown function (Fig. 6). This finding indicates that these MGEs could be used as markers to distinguish isolates of clade D from those of the other clades, and further supports the notion that the MSSA isolates from China (accounting for 91.67% of clade D isolates) evolved independently. Furthermore, we identified several clade-specific nonsynonymous SNPs associated with virulence genes, suggesting that these mutations might affect the expression of the bacterial virulence.

It is a common notion that MSSA lineages always carry a wider array of virulence genes than MRSA lineages, and that this difference is due to the fitness cost associated with methicillin resistance (28). However, we found that the number of virulence genes did not significantly differ between ST72 MRSA and MSSA isolates (median 127 versus 126, *P > *0.05), and the distributions of nearly all virulence genes (115/120) did not significantly differ between the two populations. Indeed, MRSA isolates of clade B had more virulence genes than MSSA isolates (median 129 versus 126, *P < *0.05) (Fig. 4B). It has been suggested that the transcription of the *agr* operon is repressed by the expression of β-lactam resistance (Bla/Mec system) in MRSA isolates, which prevents the production of core genome-encoded toxins (including PSMs and alpha-toxin) regulated by this operon (29). However, Chen et al. (8) showed that for the PVL-negative CA-MRSA ST72, phenol-soluble modulins (PSMs) and the global virulence regulator Agr play a key role in lysis of neutrophils and erythrocytes, while alpha-toxin and Agr strongly affect *in vivo* virulence, suggesting that the toxin regulator Agr is not significantly repressed in ST72 MRSA. As expected, nearly all ST72 isolates (97.2%, 104/107) in this study carried both PSMs and alpha-toxin (Fig. 4). Moreover, Otto reported that both expression of core genome-encoded toxins (including PSMs and alpha-toxin) and acquisition of virulence-associated genes could contribute to the evolution of CA-MRSA virulence (7). Hence, based on the results of our analysis of virulence genes, we speculate that the virulence potential of ST72 MRSA isolates is comparable to that of MSSA predecessors, and that this virulence potential is associated with expression of core genome-encoded toxins with no loss or even gain of related virulence genes that could contribute to virulence in ST72 MRSA isolates.

This study has some limitations. First, the number of ST72 isolates collected was not large enough. Second, the lack of detailed epidemiological information may have impacted the analysis of the epidemiology of ST72. Third, the findings of comparisons between isolates should be interpreted with caution, because these might be the result of differences in sampling sites and are subject to a statistical caveat, i.e., the presence and absence of several antibiotic resistance or virulence genes could not be considered independent since they are linked on the same MGEs. Further experimental evidence is needed to prove that ST72 MRSA and MSSA isolates have similar virulence.

In conclusion, our data provide important insight into the phylogenetic relationship, transmission, antibiotic resistance, and pathogenicity of the ST72 lineage on a global scale. Our results reveal that, in China, all MSSA isolates might have evolved independently of MRSA isolates from that nation. We demonstrated that ST72 isolates have the potential for international transmission as well as interregional transmission within China. Moreover, the MSSA isolates of clade D exhibited lower antibiotic resistance than isolates of the other clades, and tended to gain specific MGEs encoding specific virulence genes, such as *tsst-1*. Importantly, our findings also indicate that ST72 MRSA isolates exhibited higher antibiotic resistance than ST72 MSSA isolates, but were comparably virulent. More genomic data and experimental verification are needed to predict the epidemic potential of the ST72 lineage in countries other than South Korea.

## MATERIALS AND METHODS

### Collection of bacterial isolates.

From January 2014 to December 2019, we isolated 2,769 S. aureus strains from patients with bloodstream infections (BSI) in 18 provinces and municipalities in China. Identification of the tested strains was performed using biochemical methods and matrix-assisted laser desorption–time of flight mass spectrometry (MALDI-TOF MS) (Bruker, Bremen, Germany). Multilocus sequence typing of all 2,769 S. aureus isolates was performed by Sanger sequencing of seven housekeeping genes (including *arcC*, *aroE*, *gmk*, *glpF*, *tpi*, *yqiL*, and *pta*), after which the sequences of the housekeeping genes were submitted to the S. aureus typing database for the assignment of ST type (https://pubmlst.org/bigsdb?db=pubmlst_saureus_seqdef). Ultimately, we obtained 24 ST72 isolates among all S. aureus isolates (Table S1).

### Antimicrobial susceptibility testing.

The antimicrobial susceptibilities to 16 antimicrobial agents (oxacillin, penicillin G, sulfamethoxazole and trimethoprim, ciprofloxacin, levofloxacin, moxifloxacin, gentamicin, amikacin, erythromycin, clindamycin, tetracycline, tigecycline, rifampin, vancomycin, linezolid, and daptomycin) were determined using the agar dilution method according to standard guidelines recommended by the Clinical and Laboratory Standards Institute (CLSI, 2020). S. aureus ATCC 25923 and ATCC 29213 were used as reference strains for quality control.

### Whole-genome sequencing and genomic analysis of ST72 isolates.

Genomic DNA of S. aureus ST72 isolates was extracted using the Ezup Column Bacteria Genomic DNA purification kit (Sangon Biotech, Shanghai, China). Sequencing of the ST72 genomes was performed on a HiSeq X 10-PE150 platform (Illumina, San Diego, CA, USA). After adaptor trimming and quality filtering, the processed Illumina reads were assembled using SPAdes v3.14.1 (30) with default settings. SCC*mec* and *spa* typing were performed using the web-based SCCmecFinder (https://cge.cbs.dtu.dk/services/SCCmecFinder) and SpaFinder (https://cge.cbs.dtu.dk/services/spatyper), respectively. Potential open reading frames (ORFs) were predicted and annotated using Prokka v1.14.5 (31). Additional genomes of 83 ST72 isolates with available geographic information were downloaded from the NCBI genome database for comparison (Table S1).

### Phylogenetic analysis and Bayesian evolutionary analysis.

Snippy v4.6.0 (https://github.com/tseemann/snippy) was used to perform reference-based mapping and identify SNPs in the ST72 core genome, with the CN1 genome (GenBank accession no. CP003979) used as a reference. Gubbins v2.4.1 (32) was used to detect the recombined regions within the core genome and remove putative recombined regions. A phylogenetic tree was constructed using the core single nucleotide polymorphisms (SNPs) from the recombination-free core-genome alignment with RAxML using a GTR model and gamma correction (1,000 bootstrap replications) (33). The ancestral geographic origins of ST72 isolates were analyzed using the Bayesian binary MCMC method in RASP v4.2 (34) with 10 parallel chains of 50 million cycles. The output from Gubbins, together with the isolation dates of the isolates, was used as the input for BactDating v1.1 (35) to obtain a dated phylogeny based on Bayesian inference. The Markov chain Monte Carlo chain lengths were run for 100 million cycles, and the effective sample sizes of the model parameters mu, sigma, and alpha were all >200. The minimum spanning trees (MSTs) based on SNP data were generated using PHYLOViZ v2.0 (https://online.phyloviz.net/index).

### Identification of antimicrobial-resistance genes, virulence genes, pathogenicity islands, and prophages.

Antimicrobial-resistance (AMR) genes and virulence genes were detected using ResFinder v4.0 (https://cge.cbs.dtu.dk/services/ResFinder/) and VFanalyzer (http://www.mgc.ac.cn/cgi-bin/VFs/v5/main.cgi?func=VFanalyzer), respectively. Pathogenicity islands and prophages were identified using BLAST (https://blast.ncbi.nlm.nih.gov/Blast.cgi), with sequences of pathogenicity islands and prophages from S. aureus as the queries.

### Detection of clade-specific SNPs.

The SNPs associated with isolates of each clade were detected using Scoary v1.6.16 (36).

### Statistical analysis.

Wilcoxon rank-sum test and chi-square test or Fisher’s exact test were used for statistical analysis, and *P* < 0.05 was considered as statistically significant. All data were analyzed using the R software (https://www.r-project.org/).

### Data availability.

The whole-genome sequences of 24 ST72 S. aureus isolates have been deposited in the GenBank database under BioProject accession no. PRJNA735348.
