# Supplementary material for: Comparative Genomic Analysis Provides Insights into the Evolution and Genetic Diversity of Community-Genotype Sequence Type 72 Staphylococcus aureus Isolates
Source: mSystems. 2021 Sep 7;6(5):e00986-21. doi: 10.1128/mSystems.00986-21 (PMC8547429; doi:10.1128/mSystems.00986-21)
Supplement: TABLE S1 [file msystems.00986-21-st001.docx]

Table S1. Information of ST72 isolates analyzed in this study.

| Strain | GenBank assembly accession | Contigs | Length | N50 | Year of isolation | Country | Host | Source of isolation | Class | spa type | SCCmec type |
| --- | --- | --- | --- | --- | --- | --- | --- | --- | --- | --- | --- |
| CN1 | GCA_000463055.1 | 3 | 2757070 | 2751266 | 2006 | South Korea | Human | Skin | MRSA | t324 | SCCmec IVc(2B) |
| M0139 | GCA_000531015.1 | 11 | 2738036 | 2217153 | 2003 | USA | Human | Blood | MSSA | t2453 | MSSA |
| HST-084 | GCA_000564955.1 | 36 | 2715285 | 258877 | 2011 | Lebanon | Human | NA | MRSA | t9831 | SCCmec V(5C2&5) |
| MRSA_CVM43477 | GCA_000830555.1 | 197 | 2756796 | 28199 | 2011 | USA | Animal | Animal | MRSA | t126 | SCCmec IVa(2B) |
| V040 | GCA_001184335.2 | 33 | 2727097 | 175443 | 2014 | South Korea | Human | Wound | MRSA | t148 | SCCmec IVc(2B) |
| TMUS2126 | GCA_001549655.1 | 1 | 2770164 | 2770164 | NA | Japan | Human | NA | MRSA | t324 | SCCmec IVc(2B) |
| TMUS2134 | GCA_001549675.1 | 1 | 2770164 | 2770164 | NA | Japan | Human | NA | MRSA | t324 | SCCmec IVc(2B) |
| FORC_012 | GCA_001580495.1 | 1 | 2815416 | 2815416 | 2009 | South Korea | Human | Respiratory tract | MRSA | t664 | SCCmec IVc(2B) |
| 2148.N | GCA_001717665.2 | 2 | 2752262 | 2748803 | 2011 | USA | Human | Respiratory tract | MSSA | t148 | MSSA |
| HMSC58B01 | GCA_001815835.1 | 85 | 2738187 | 108968 | NA | USA | Human | Wound | MSSA | t4359 | MSSA |
| SA_70002 | GCA_001900005.1 | 31 | 2679548 | 290256 | 2014 | Spain | Human | Blood | MRSA | t148 | SCCmec IVc(2B) |
| SA_190006 | GCA_001900165.1 | 78 | 2765439 | 272991 | 2013 | Spain | Human | Blood | MSSA | NA | MSSA |
| CFSA157 | GCA_002123615.1 | 31 | 2710250 | 435420 | 2014 | USA | Human | Respiratory tract | MSSA | t1346 | MSSA |
| CFSA012 | GCA_002125915.1 | 53 | 2750260 | 199620 | 2014 | USA | Human | Respiratory tract | MRSA | t126 | SCCmec IVa(2B) |
| RB210 | GCA_002141045.1 | 9 | 2783870 | 681155 | 2016 | USA | Human | NA | MSSA | t148 | MSSA |
| FORC_040 | GCA_002214665.1 | 2 | 2764958 | 2760844 | 2013 | South Korea | Animal | Animal | MRSA | t324 | SCCmec IVc(2B) |
| ABKWS | GCA_002221895.1 | 77 | 2742009 | 86874 | 2015 | USA | Human | NA | MSSA | t537 | MSSA |
| TCH130 | GCA_000159555.1 | 248 | 2779997 | 418208 | NA | USA | Human | NA | MSSA | t6509 | MSSA |
| UE200 | GCA_002267215.1 | 61 | 2720583 | 86461 | 2012 | Ecuador | Human | Blood | MRSA | t605 | SCCmec IVc(2B) |
| UA813 | GCA_002267785.1 | 50 | 2719465 | 105798 | 2013 | Argentina | Human | Blood | MRSA | t605 | SCCmec IVc(2B) |
| UP1006 | GCA_002887475.1 | 77 | 2733240 | 89651 | 2013 | Peru | Human | Blood | MRSA | NA | SCCmec IVc(2B) |
| E16SA093 | GCA_003342735.1 | 1 | 2785564 | 2785564 | 2016 | South Korea | Human | Blood | MRSA | t324 | NA |
| F17SA003 | GCA_003342775.1 | 1 | 2768346 | 2768346 | 2017 | South Korea | Human | Blood | MRSA | NA | NA |
| FORC_061 | GCA_003347055.1 | 2 | 2749621 | 2731682 | 2017 | South Korea | Human | Blood | MSSA | t126 | MSSA |
| COAS6020 | GCA_000540635.1 | 36 | 2730192 | 147022 | NA | USA | Human | NA | MRSA | NA | SCCmec IVc(2B) |
| UCIM6080 | GCA_000558805.1 | 33 | 2753263 | 157287 | NA | USA | Human | Blood | MRSA | NA | SCCmec IVa(2B) |
| BCH-SA-14 | GCA_003720845.1 | 36 | 2733025 | 578911 | 2018 | USA | Human | Respiratory tract | MSSA | t1346 | MSSA |
| BCH-SA-03 | GCA_003721095.1 | 47 | 2695371 | 443662 | 2018 | USA | Human | Respiratory tract | MRSA | t9602 | SCCmec IVc(2B) |
| ER03364.3 | GCA_008936655.1 | 1 | 2786143 | 2786143 | 2015 | USA | Human | Blood | MRSA | t148 | SCCmec V(5C2&5) |
| MRSA703 | GCA_009457185.1 | 32 | 2724049 | 213580 | 2016 | USA | Human | Respiratory tract | MRSA | t324 | SCCmec IVc(2B) |
| MRSA517 | GCA_009457775.1 | 31 | 2739909 | 193778 | 2016 | USA | Human | Respiratory tract | MRSA | t779 | SCCmec IVg(2B) |
| MRSA505 | GCA_009457995.1 | 41 | 2783503 | 174478 | 2016 | USA | Human | Wound | MRSA | t7098 | SCCmec IVa(2B) |
| MRSA97 | GCA_009458205.1 | 27 | 2731544 | 234329 | 2016 | USA | Human | NA | MRSA | t148 | SCCmec IV*(2B) |
| MRSA297 | GCA_009459815.1 | 38 | 2793766 | 185500 | 2016 | USA | Human | Respiratory tract | MRSA | t148 | SCCmec IVa(2B) |
| MRSA298 | GCA_009459745.1 | 56 | 2867339 | 109627 | 2016 | USA | Human | Respiratory tract | MRSA | t126 | SCCmec IVa(2B) |
| MRSA285 | GCA_009460035.1 | 32 | 2741201 | 176733 | 2016 | USA | Human | Respiratory tract | MRSA | t2703 | SCCmec IVa(2B) |
| MRSA276 | GCA_009460215.1 | 32 | 2740467 | 171162 | 2016 | USA | Human | Respiratory tract | MRSA | t2703 | SCCmec IVa(2B) |
| UA885 | GCA_009674915.1 | 82 | 2746698 | 210888 | 2013 | Argentina | Human | Blood | MSSA | t1346 | MSSA |
| UA850 | GCA_009675735.1 | 80 | 2701256 | 83601 | 2013 | Argentina | Human | Blood | MSSA | t3092 | MSSA |
| UA919 | GCA_009675965.1 | 78 | 2726728 | 207245 | 2013 | Argentina | Human | Blood | MSSA | NA | MSSA |
| 5173 | GCA_010569345.1 | 52 | 2755863 | 172046 | 2018 | Colombia | Human | Blood | MSSA | t4100 | MSSA |
| UMX758 | GCA_011023445.1 | 81 | 2702871 | 78294 | 2012 | Mexico | Human | Blood | MSSA | t5574 | MSSA |
| UC743 | GCA_011023645.1 | 164 | 2701130 | 37324 | 2013 | Colombia | Human | Blood | MSSA | t7093 | MSSA |
| UG1037 | GCA_011024525.1 | 234 | 2698414 | 22682 | 2013 | Guatemala | Human | Blood | MSSA | t148 | MSSA |
| UG941 | GCA_011024755.1 | 109 | 2761645 | 59042 | 2013 | Guatemala | Human | Blood | MSSA | t148 | MSSA |
| UC732 | GCA_011025725.1 | 85 | 2716142 | 70032 | 2013 | Colombia | Human | Blood | MSSA | t7093 | MSSA |
| UG262 | GCA_011027445.1 | 323 | 2654129 | 14129 | 2012 | Guatemala | Human | Blood | MSSA | t148 | MSSA |
| UG263 | GCA_011027565.1 | 282 | 2672624 | 19103 | 2012 | Guatemala | Human | Blood | MSSA | t148 | MSSA |
| UC21 | GCA_011031255.1 | 83 | 2761485 | 74937 | 2011 | Colombia | Human | Blood | MSSA | t148 | MSSA |
| UC20 | GCA_011031695.1 | 91 | 2765928 | 54220 | 2011 | Colombia | Human | Blood | MSSA | t148 | MSSA |
| UP1021 | GCA_011061085.1 | 73 | 2758287 | 101870 | NA | Peru | Human | Blood | MRSA | t148 | SCCmec IVc(2B) |
| UA866 | GCA_011777445.1 | 32 | 2741587 | 272972 | NA | Argentina | Human | Blood | MRSA | t148 | SCCmec IVc(2B) |
| CV581 | GCA_012651265.1 | 32 | 2756757 | 247108 | 2014 | Cape Verde | Human | Respiratory tract | MSSA | t148 | MSSA |
| CV274 | GCA_012653005.1 | 26 | 2748773 | 333116 | 2013 | Cape Verde | Human | Respiratory tract | MSSA | t148 | MSSA |
| MRSA297_6GL | GCA_013337965.1 | 35 | 2788735 | 267802 | 2016 | USA | Clinical environment | Clinical environment | MRSA | t148 | SCCmec IVa(2B) |
| MRSA703_10GL | GCA_013338405.1 | 32 | 2721591 | 200887 | 2016 | USA | Clinical environment | Clinical environment | MRSA | t324 | SCCmec IVc(2B) |
| pt290 | GCA_014725935.1 | 1 | 2854231 | 2854231 | 2017 | USA | Human | Blood | MRSA | t1597 | SCCmec IVa(2B) |
| pt252 | GCA_014726555.1 | 1 | 2759292 | 2759292 | 2016 | USA | Human | Blood | MRSA | t664 | SCCmec IVc(2B) |
| ER11011.3 | GCA_014730635.1 | 1 | 2860401 | 2860401 | 2017 | USA | Human | Blood | MRSA | t148 | SCCmec IVa(2B) |
| ER10823.3 | GCA_014730675.1 | 1 | 2865914 | 2865914 | 2017 | USA | Human | Blood | MRSA | t7098 | SCCmec IVa(2B) |
| ER10617.3 | GCA_014730815.1 | 1 | 2867545 | 2867545 | 2017 | USA | Human | Blood | MRSA | t148 | SCCmec IVa(2B) |
| ER11455.3A | GCA_014734015.1 | 2 | 2870203 | 2809032 | 2017 | USA | Human | Blood | MRSA | t2473 | SCCmec IVa(2B) |
| AUSMDU00008431 | GCA_015600245.1 | 102 | 2775233 | 60273 | 2017 | Australia | Human | NA | MRSA | t4359 | SCCmec IVc(2B) |
| VL-FL-7270 | GCA_015610065.1 | 36 | 2757710 | 162517 | 2017 | USA | Animal | Animal | MRSA | NA | SCCmec IVa(2B) |
| MRSA10251 | GCA_015712295.1 | 29 | 2687973 | 254713 | 2017 | USA | Human | Respiratory tract | MRSA | t126 | SCCmec IVa(2B) |
| 119-683 | GCA_015712905.1 | 24 | 2781684 | 276624 | 2014 | USA | Human | Skin | MSSA | t148 | MSSA |
| 81-904 | GCA_015713605.1 | 20 | 2793112 | 338213 | 2013 | USA | Human | Skin | MSSA | t148 | MSSA |
| 39-367 | GCA_015714225.1 | 22 | 2747182 | 446525 | 2012 | USA | Human | Skin | MSSA | t148 | MSSA |
| MRSA10217 | GCA_015715295.1 | 32 | 2716254 | 214823 | 2017 | USA | Human | Wound | MRSA | NA | SCCmec IVa(2B) |
| MRSA10390 | GCA_015726605.1 | 35 | 2713982 | 199392 | 2018 | USA | Human | Respiratory tract | MRSA | t2703 | SCCmec IVa(2B) |
| MRSA10312 | GCA_015728175.1 | 27 | 2732271 | 271462 | 2017 | USA | Human | Blood | MRSA | t4359 | SCCmec IVa(2B) |
| MRSA10595 | GCA_017154655.1 | 22 | 2720613 | 381871 | 2019 | USA | Human | Respiratory tract | MRSA | t148 | SCCmec IV*(2B) |
| MRSA10545 | GCA_017156035.1 | 31 | 2795231 | 321996 | 2019 | USA | Human | Blood | MRSA | t1597 | SCCmec IVa(2B) |
| 6105 | GCA_017581645.1 | 16 | 2710234 | 257438 | 2013 | France | Human | Blood | MSSA | t537 | MSSA |
| IHMA92 | GCA_017695565.1 | 54 | 2787885 | 181976 | 2016 | USA | Human | Eye | MRSA | t126 | SCCmec IVa(2B) |
| IHMA38 | GCA_017698015.1 | 42 | 2720141 | 273080 | 2014 | USA | Human | Eye | MSSA | t148 | MSSA |
| 3688STDY6124895 | GCA_900125915.1 | 16 | 2748329 | 593811 | 2015 | Thailand | Human | NA | MSSA | t148 | MSSA |
| 3688STDY6124944 | GCA_900126365.1 | 19 | 2719434 | 465150 | 2015 | Thailand | Human | NA | MSSA | t537 | MSSA |
| CHUV_12 | GCA_900155825.1 | 39 | 2847575 | 204215 | 2014 | Switzerland | Human | Skin | MRSA | t3169 | SCCmec IVa(2B) |
| H1356 | GCA_900250315.1 | 37 | 2809218 | 302469 | 2014 | Denmark | Human | NA | MRSA | t791 | SCCmec IVa(2B) |
| M3140 | GCA_900251275.1 | 89 | 2845501 | 98714 | 2014 | Denmark | Human | NA | MRSA | t791 | SCCmec IVa(2B) |
| K07-204 | GCA_014349095.1 | 49 | 2737995 | 288898 | 2007 | South Korea | Human | NA | MRSA | t664 | SCCmec IVc(2B) |
| K07-561 | GCA_014297235.1 | 39 | 2720222 | 177039 | 2007 | South Korea | Human | NA | MSSA | t126 | MSSA |
| SKLX58369 | This study | 48 | 2732853 | 283642 | 2016 | China | Human | Blood | MRSA | t664 | SCCmec IVc(2B) |
| SKLX83971 | This study | 44 | 2730364 | 289005 | 2018 | China | Human | Blood | MRSA | t324 | SCCmec IVc(2B) |
| SKLX83977 | This study | 46 | 2730588 | 289005 | 2018 | China | Human | Blood | MRSA | t324 | SCCmec IVc(2B) |
| SKLX60471 | This study | 52 | 2738395 | 181640 | 2017 | China | Human | Blood | MRSA | t324 | SCCmec IVc(2B) |
| SKLX94718 | This study | 39 | 2734577 | 289221 | 2019 | China | Human | Blood | MRSA | t2431 | SCCmec IVc(2B) |
| SKLX109490 | This study | 44 | 2732011 | 288939 | 2019 | China | Human | Blood | MRSA | t2431 | SCCmec IVc(2B) |
| SKLX115188 | This study | 39 | 2731391 | 289329 | 2019 | China | Human | Blood | MRSA | t2431 | SCCmec IVc(2B) |
| SKLX115236 | This study | 39 | 2731471 | 289329 | 2019 | China | Human | Blood | MRSA | t2431 | SCCmec IVc(2B) |
| SKLX55661 | This study | 45 | 2731626 | 288805 | 2015 | China | Human | Blood | MRSA | t2461 | SCCmec IVc(2B) |
| SKLX56774 | This study | 38 | 2755705 | 371760 | 2016 | China | Human | Blood | MRSA | t148 | SCCmec V(5C2&5) |
| SKLX56776 | This study | 39 | 2756031 | 269849 | 2016 | China | Human | Blood | MRSA | t148 | SCCmec V(5C2&5) |
| SKLX56854 | This study | 39 | 2772636 | 268006 | 2016 | China | Human | Blood | MRSA | t4100 | SCCmec V(5C2&5) |
| SKLX79556 | This study | 41 | 2773185 | 225612 | 2017 | China | Human | Blood | MRSA | t148 | SCCmec V(5C2&5) |
| SKLX101809 | This study | 33 | 2758539 | 303620 | 2019 | China | Human | Blood | MSSA | t901 | MSSA |
| SKLX58678 | This study | 28 | 2719221 | 336220 | 2016 | China | Human | Blood | MSSA | t148 | MSSA |
| SKLX67499 | This study | 29 | 2735476 | 233461 | 2017 | China | Human | Blood | MSSA | t148 | MSSA |
| SKLX88606 | This study | 32 | 2732951 | 336663 | 2019 | China | Human | Blood | MSSA | t17071 | MSSA |
| SKLX100388 | This study | 30 | 2740169 | 336333 | 2019 | China | Human | Blood | MSSA | t148 | MSSA |
| SKLX117421 | This study | 63 | 2692232 | 89599 | 2019 | China | Human | Blood | MSSA | t148 | MSSA |
| SKLX117479 | This study | 31 | 2735388 | 335165 | 2019 | China | Human | Blood | MSSA | t3735 | MSSA |
| SKLX54748 | This study | 32 | 2737266 | 335406 | 2015 | China | Human | Blood | MSSA | NA | MSSA |
| SKLX52559 | This study | 36 | 2727213 | 358259 | 2015 | China | Human | Blood | MSSA | t148 | MSSA |
| SKLX51500 | This study | 30 | 2751917 | 305173 | 2014 | China | Human | Blood | MSSA | t3169 | MSSA |
| SKLX53761 | This study | 27 | 2676479 | 358219 | 2015 | China | Human | Blood | MSSA | t452 | MSSA |

NA, not available.
