# Supplementary figures and images for: Comparative Genomic Analysis Provides Insights into the Evolution and Genetic Diversity of Community-Genotype Sequence Type 72 Staphylococcus aureus Isolates
Source: mSystems. 2021 Sep 7;6(5):e00986-21. doi: 10.1128/mSystems.00986-21 (PMC8547429; doi:10.1128/mSystems.00986-21)

A

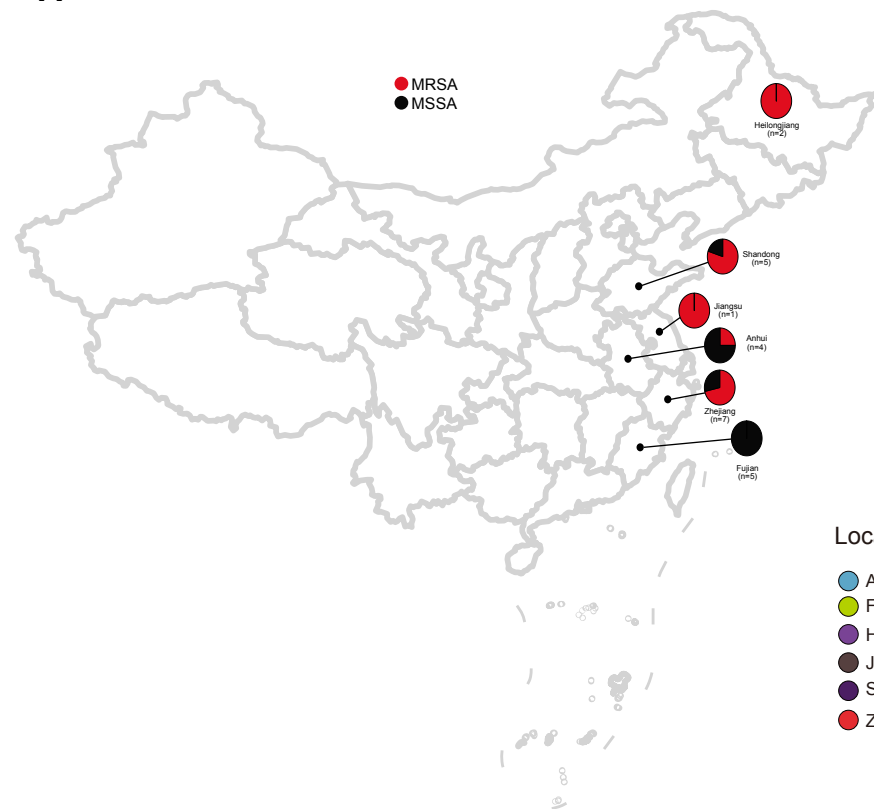

C

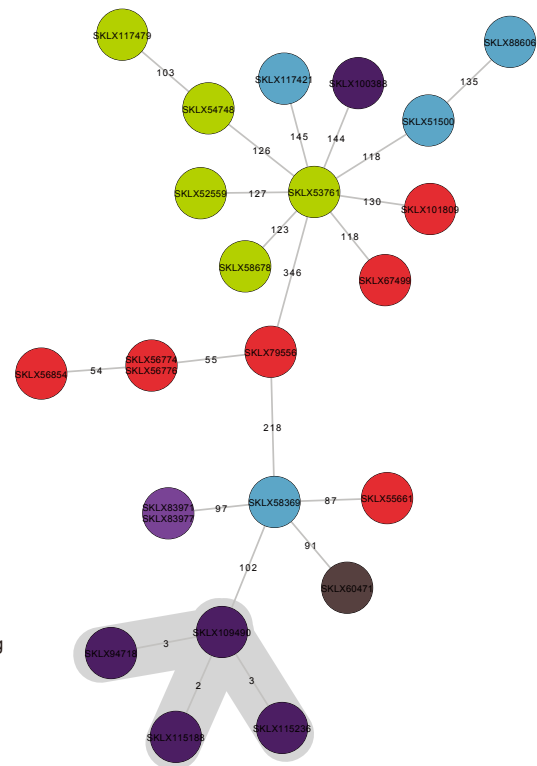

B

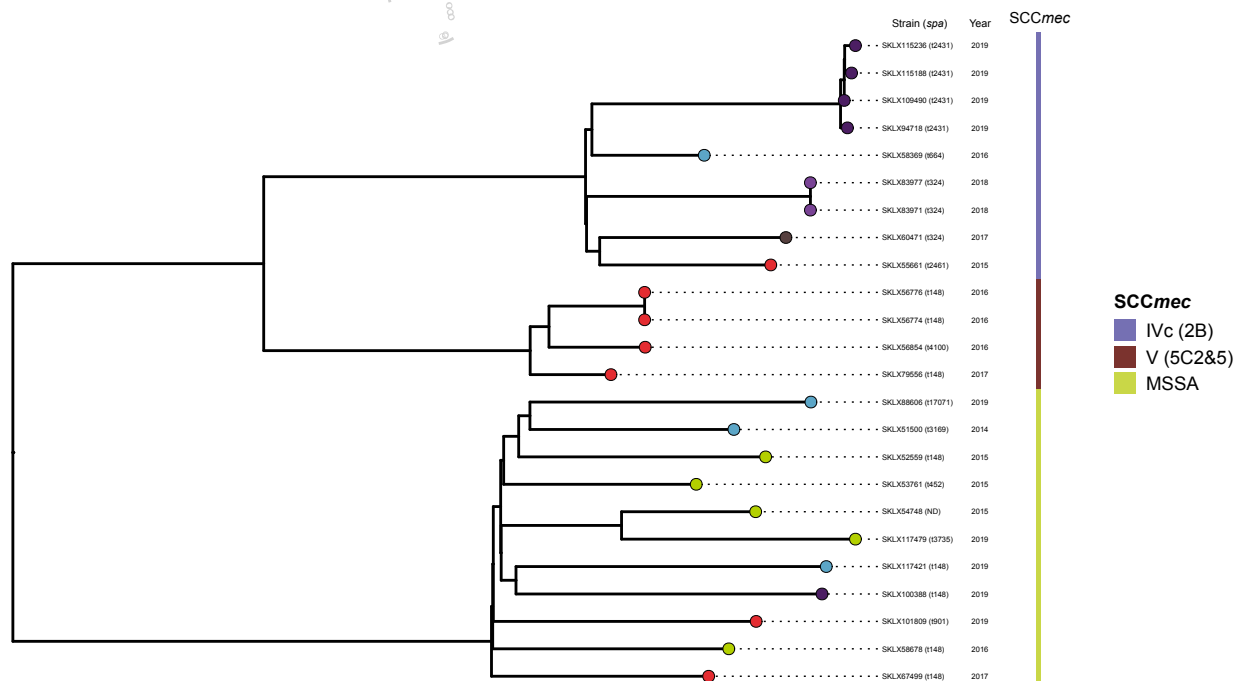

Supplement: FIG S1 [file msystems.00986-21-sf001.pdf]

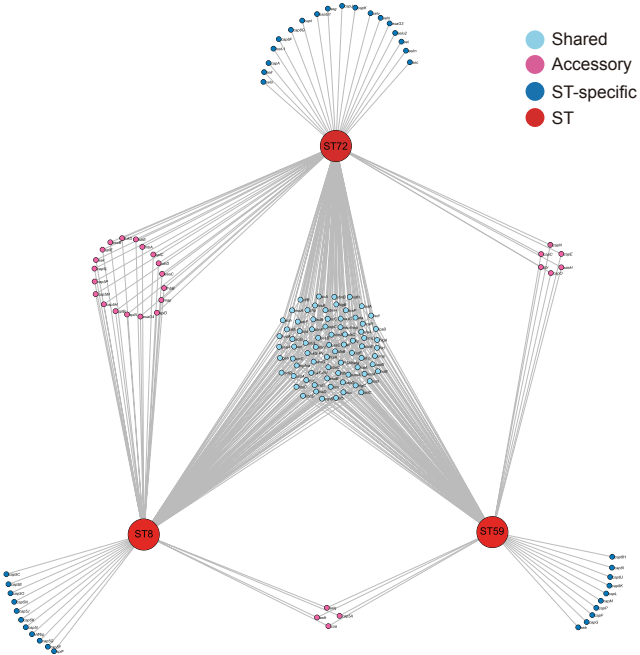

Supplement: FIG S2 [file msystems.00986-21-sf002.pdf]

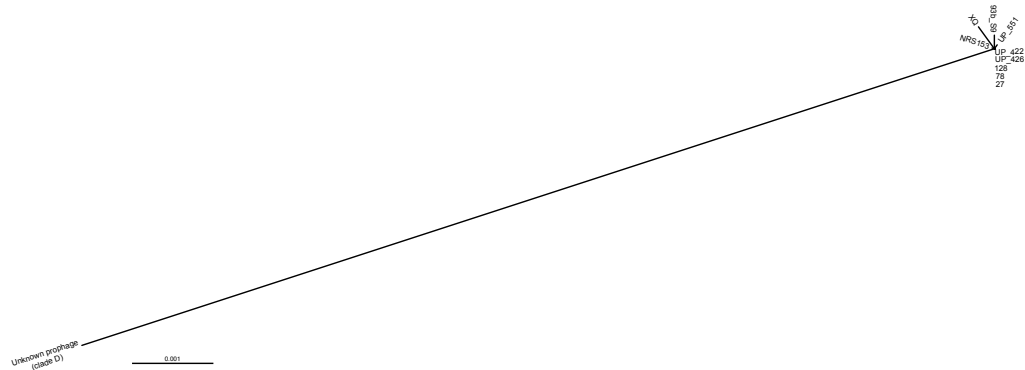

Supplement: FIG S3 [file msystems.00986-21-sf003.pdf]

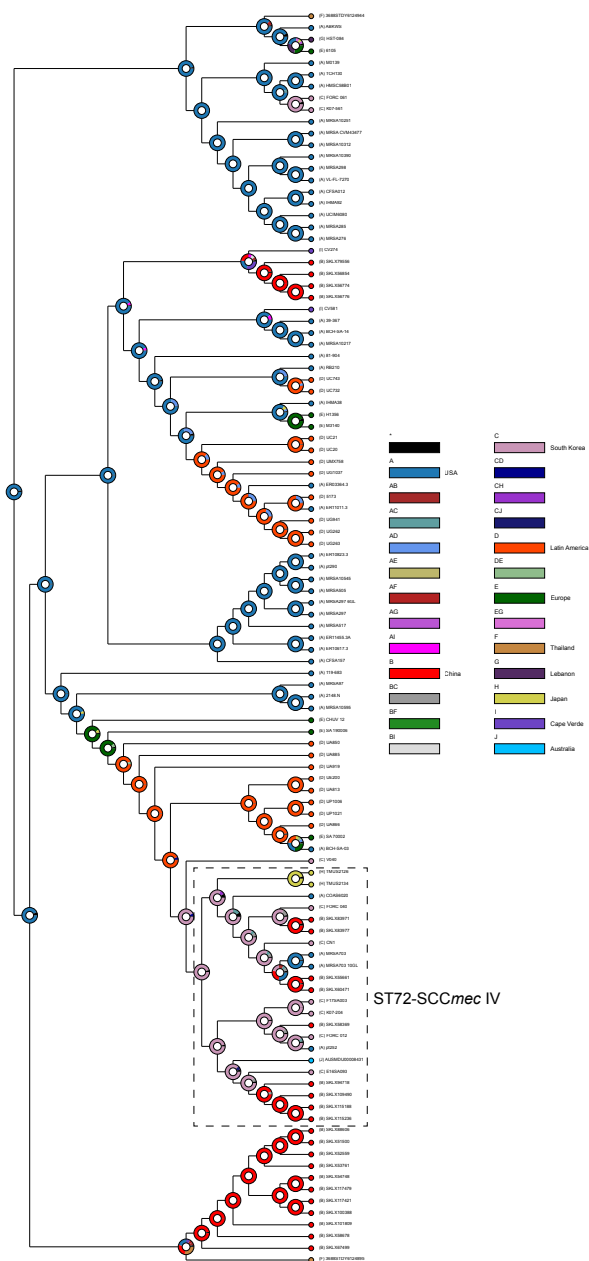

Supplement: FIG S4 [file msystems.00986-21-sf004.pdf]
