# Supplementary material for: Comparative Genomic Analysis Provides Insights into the Evolution and Genetic Diversity of Community-Genotype Sequence Type 72 Staphylococcus aureus Isolates
Source: mSystems. 2021 Sep 7;6(5):e00986-21. doi: 10.1128/mSystems.00986-21 (PMC8547429; doi:10.1128/mSystems.00986-21)
Supplement: TABLE S2 [file msystems.00986-21-st002.docx]

Table S2. Distributions of core virulence genes in the ST72 lineage (≥85% isolates per clade).

| Category | Virulence gene | No. (%) of isolates | | | |
| --- | --- | --- | --- | --- | --- |
|  |  | Clade A (*n* = 20) | Clade B (*n* = 36) | Clade C (*n* = 39) | Clade D (*n* = 12) |
| Adherence | *atl* | 20 (100) | 36 (100) | 39 (100) | 12 (100) |
|  | *eap/map* | 20 (100) | 35 (97.22) | 39 (100) | 12 (100) |
|  | *ebp* | 19 (95) | 36 (100) | 39 (100) | 12 (100) |
|  | *efb* | 20 (100) | 36 (100) | 39 (100) | 12 (100) |
|  | *emp* | 20 (100) | 36 (100) | 39 (100) | 12 (100) |
|  | *fnbA* | 20 (100) | 33 (91.67) | 39 (100) | 12 (100) |
|  | *fnbB* | 19 (95) | 34 (94.44) | 39 (100) | 12 (100) |
|  | *icaA* | 20 (100) | 35 (97.22) | 39 (100) | 12 (100) |
|  | *icaB* | 20 (100) | 36 (100) | 39 (100) | 12 (100) |
|  | *icaC* | 20 (100) | 36 (100) | 39 (100) | 12 (100) |
|  | *icaD* | 20 (100) | 36 (100) | 39 (100) | 12 (100) |
|  | *icaR* | 20 (100) | 35 (97.22) | 39 (100) | 12 (100) |
|  | *sasC* | 19 (95) | 36 (100) | 39 (100) | 12 (100) |
|  | *sasH* | 20 (100) | 36 (100) | 39 (100) | 12 (100) |
|  | *spa* | 20 (100) | 36 (100) | 39 (100) | 12 (100) |
| Exoenzyme | *aur* | 20 (100) | 36 (100) | 39 (100) | 12 (100) |
|  | *geh* | 20 (100) | 36 (100) | 39 (100) | 12 (100) |
|  | *hysA* | 20 (100) | 36 (100) | 39 (100) | 12 (100) |
|  | *lip* | 20 (100) | 36 (100) | 39 (100) | 12 (100) |
|  | *nuc* | 20 (100) | 36 (100) | 39 (100) | 12 (100) |
|  | *sak* | 17 (85) | 32 (88.89) | 35 (89.74) | 11 (91.67) |
|  | *splA* | 20 (100) | 31 (86.11) | 39 (100) | 12 (100) |
|  | *splB* | 20 (100) | 31 (86.11) | 38 (97.44) | 12 (100) |
|  | *splC* | 20 (100) | 31 (86.11) | 38 (97.44) | 12 (100) |
|  | *splD* | 19 (95) | 31 (86.11) | 39 (100) | 12 (100) |
|  | *sspA* | 20 (100) | 36 (100) | 39 (100) | 12 (100) |
|  | *sspB* | 20 (100) | 36 (100) | 39 (100) | 12 (100) |
|  | *sspC* | 20 (100) | 36 (100) | 39 (100) | 12 (100) |
| Immune evasion | *cap5H* | 20 (100) | 36 (100) | 39 (100) | 12 (100) |
|  | *cap5L* | 20 (100) | 36 (100) | 38 (97.44) | 12 (100) |
|  | *cap5M* | 20 (100) | 36 (100) | 39 (100) | 12 (100) |
|  | *cap5P* | 20 (100) | 36 (100) | 39 (100) | 12 (100) |
|  | *cap8F* | 20 (100) | 36 (100) | 39 (100) | 12 (100) |
|  | *cap8G* | 20 (100) | 36 (100) | 39 (100) | 12 (100) |
|  | *capA* | 20 (100) | 35 (97.22) | 39 (100) | 12 (100) |
|  | *capB* | 20 (100) | 36 (100) | 39 (100) | 12 (100) |
|  | *capC* | 20 (100) | 36 (100) | 39 (100) | 12 (100) |
|  | *capD* | 20 (100) | 36 (100) | 39 (100) | 12 (100) |
|  | *capE* | 20 (100) | 36 (100) | 39 (100) | 12 (100) |
|  | *capI* | 20 (100) | 36 (100) | 38 (97.44) | 12 (100) |
|  | *capJ* | 20 (100) | 36 (100) | 38 (97.44) | 12 (100) |
|  | *capK* | 20 (100) | 36 (100) | 38 (97.44) | 12 (100) |
|  | *capN* | 19 (95) | 36 (100) | 39 (100) | 12 (100) |
|  | *capO* | 20 (100) | 36 (100) | 39 (100) | 12 (100) |
|  | *sbi* | 19 (95) | 36 (100) | 39 (100) | 12 (100) |
|  | *scn* | 17 (85) | 32 (88.89) | 35 (89.74) | 11 (91.67) |
| Secretion system | *esaA* | 20 (100) | 36 (100) | 39 (100) | 12 (100) |
|  | *esaB* | 20 (100) | 36 (100) | 39 (100) | 12 (100) |
|  | *esaC* | 20 (100) | 36 (100) | 39 (100) | 12 (100) |
|  | *esaG1* | 17 (85) | 33 (91.67) | 39 (100) | 12 (100) |
|  | *essA* | 20 (100) | 36 (100) | 39 (100) | 12 (100) |
|  | *essB* | 20 (100) | 36 (100) | 39 (100) | 12 (100) |
|  | *essC* | 20 (100) | 36 (100) | 39 (100) | 12 (100) |
|  | *esxA* | 20 (100) | 36 (100) | 39 (100) | 12 (100) |
|  | *esxB* | 20 (100) | 36 (100) | 39 (100) | 12 (100) |
| Enterotoxin | *seg* | 19 (95) | 33 (91.67) | 39 (100) | 11 (91.67) |
|  | *sei* | 20 (100) | 32 (88.89) | 39 (100) | 11 (91.67) |
|  | *selm* | 19 (95) | 34 (94.44) | 39 (100) | 11 (91.67) |
|  | *seln* | 20 (100) | 33 (91.67) | 39 (100) | 11 (91.67) |
|  | *selo* | 20 (100) | 34 (94.44) | 35 (89.74) | 12 (100) |
|  | *selu2* | 19 (95) | 33 (91.67) | 39 (100) | 12 (100) |
| Exotoxin | *set* | 20 (100) | 36 (100) | 39 (100) | 12 (100) |
| Exfoliative toxin | *eta* | 20 (100) | 36 (100) | 39 (100) | 12 (100) |
| Leukocidin | *lukD* | 20 (100) | 36 (100) | 39 (100) | 11 (91.67) |
|  | *lukE* | 20 (100) | 36 (100) | 39 (100) | 11 (91.67) |
| Phenol-soluble modulin | *psmA* | 20 (100) | 36 (100) | 39 (100) | 12 (100) |
|  | *PSMbeta* | 20 (100) | 35 (97.22) | 39 (100) | 12 (100) |
| Hemolysin | *hlb* | 20 (100) | 36 (100) | 39 (100) | 12 (100) |
|  | *hld* | 20 (100) | 34 (94.44) | 39 (100) | 12 (100) |
|  | *hlgA* | 20 (100) | 35 (97.22) | 39 (100) | 12 (100) |
|  | *hlgB* | 20 (100) | 36 (100) | 39 (100) | 12 (100) |
|  | *hlgC* | 20 (100) | 36 (100) | 39 (100) | 12 (100) |
|  | *hly/hla* | 20 (100) | 35 (97.22) | 37 (94.87) | 12 (100) |
| Iron uptake | *htsA* | 20 (100) | 36 (100) | 39 (100) | 12 (100) |
|  | *htsB* | 19 (95) | 36 (100) | 39 (100) | 12 (100) |
|  | *htsC* | 20 (100) | 36 (100) | 39 (100) | 12 (100) |
|  | *isdA* | 20 (100) | 36 (100) | 39 (100) | 12 (100) |
|  | *isdB* | 20 (100) | 36 (100) | 39 (100) | 12 (100) |
|  | *isdC* | 20 (100) | 36 (100) | 39 (100) | 12 (100) |
|  | *isdD* | 20 (100) | 36 (100) | 39 (100) | 12 (100) |
|  | *isdE* | 20 (100) | 36 (100) | 39 (100) | 12 (100) |
|  | *isdF* | 20 (100) | 36 (100) | 39 (100) | 12 (100) |
|  | *isdG* | 20 (100) | 36 (100) | 39 (100) | 12 (100) |
|  | *isdH* | 19 (95) | 36 (100) | 39 (100) | 12 (100) |
|  | *isdI* | 20 (100) | 36 (100) | 39 (100) | 12 (100) |
|  | *sbnA* | 20 (100) | 36 (100) | 39 (100) | 12 (100) |
|  | *sbnB* | 20 (100) | 36 (100) | 39 (100) | 12 (100) |
|  | *sbnC* | 20 (100) | 36 (100) | 39 (100) | 12 (100) |
|  | *sbnD* | 20 (100) | 36 (100) | 39 (100) | 12 (100) |
|  | *sbnE* | 20 (100) | 36 (100) | 39 (100) | 12 (100) |
|  | *sbnF* | 20 (100) | 36 (100) | 39 (100) | 12 (100) |
|  | *sbnG* | 20 (100) | 36 (100) | 39 (100) | 12 (100) |
|  | *sbnH* | 20 (100) | 36 (100) | 39 (100) | 12 (100) |
|  | *sbnI* | 20 (100) | 36 (100) | 39 (100) | 12 (100) |
|  | *sfaA* | 20 (100) | 36 (100) | 39 (100) | 12 (100) |
|  | *sfaB* | 20 (100) | 36 (100) | 39 (100) | 12 (100) |
|  | *sfaC* | 20 (100) | 36 (100) | 39 (100) | 12 (100) |
|  | *sfaD* | 20 (100) | 36 (100) | 39 (100) | 12 (100) |
|  | *sirA* | 20 (100) | 36 (100) | 39 (100) | 12 (100) |
|  | *sirB* | 20 (100) | 36 (100) | 39 (100) | 12 (100) |
|  | *sirC* | 20 (100) | 36 (100) | 39 (100) | 12 (100) |
|  | *srtB* | 20 (100) | 33 (91.67) | 39 (100) | 12 (100) |
