# Supplementary material for: Comparative Genomic Analysis Provides Insights into the Evolution and Genetic Diversity of Community-Genotype Sequence Type 72 Staphylococcus aureus Isolates
Source: mSystems. 2021 Sep 7;6(5):e00986-21. doi: 10.1128/mSystems.00986-21 (PMC8547429; doi:10.1128/mSystems.00986-21)
Supplement: TABLE S3 [file msystems.00986-21-st003.docx]

Table S3. Clade-specific SNPs as identified by Scoary

| Clade | LOCUS_TAG | STRAND | NT_POS^a^ | AA_POS^b^ | EFFECT | GENE | PRODUCT |
| --- | --- | --- | --- | --- | --- | --- | --- |
| A | SAKOR_00016 | + | 281/1401 | 94/466 | missense_variant c.281T>C p.Ile94Thr |  | Replicative DNA helicase |
|  | SAKOR_00132 | + | 1592/1917 | 531/638 | missense_variant c.1592A>G p.Asp531Gly | *capD* | capsular polysaccharide synthesis enzyme CapD |
|  | SAKOR_00157 | + | 220/1176 | 74/391 | missense_variant c.220G>A p.Ala74Thr |  | D-dependent formate dehydrogenase |
|  | SAKOR_00157 | + | 910/1176 | 304/391 | missense_variant c.910G>A p.Val304Ile |  | D-dependent formate dehydrogenase |
|  | SAKOR_00224 | + | 289/1260 | 97/419 | missense_variant c.289G>A p.Val97Ile |  | PTS system, galactitol-specific IIC component |
|  | SAKOR_00243 | - | 523/705 | 175/234 | missense_variant c.523A>G p.Asn175Asp |  | Transcriptional regulator, GntR family protein |
|  | SAKOR_00250 | - | 736/999 | 246/332 | missense_variant c.736T>G p.Ser246Ala |  | Transcriptional repressor |
|  | SAKOR_00275 | + | 653/1854 | 218/617 | missense_variant c.653A>C p.Glu218Ala |  | Putative cytosolic protein |
|  | SAKOR_00453 | - | 314/1119 | 105/372 | missense_variant c.314G>A p.Gly105Glu |  | Putative membrane spanning protein |
|  | SAKOR_00506 | - | 195/1215 | 65/404 | synonymous_variant c.195C>T p.Tyr65Tyr |  | Nucleoside transporter |
|  | SAKOR_00657 | + | 257/1902 | 86/633 | missense_variant c.257G>A p.Arg86Gln | *varG* | ABC transporter permease protein VraG |
|  | SAKOR_00790 | + | 548/2877 | 183/958 | missense_variant c.548C>A p.Pro183His | *clfA* | Fibronectin-binding protein |
|  | SAKOR_00840 | + | 355/780 | 119/259 | missense_variant c.355A>G p.Ile119Val |  | Hydrolase (HAD superfamily) |
|  | SAKOR_00963 | + | 854/1371 | 285/456 | missense_variant c.854A>C p.Glu285Ala |  | Isochorismate synthase |
|  | SAKOR_00974 | - | 1273/3792 | 425/1263 | missense_variant c.1273G>C p.Gly425Arg | *atl* | Peptidoglycan endo-beta-N-acetylglucosaminidase |
|  | SAKOR_00975 | - | 253/450 | 85/149 | stop_gained c.253C>T p.Gln85* |  | Acetyltransferase, GT family |
|  | SAKOR_01007 | + | 1040/1362 | 347/453 | missense_variant c.1040A>T p.Lys347Ile |  | Cytochrome d ubiquinol oxidase subunit I |
|  | SAKOR_01128 | + | 755/1137 | 252/378 | missense_variant c.755G>A p.Gly252Glu |  | Carbamoyl-phosphate synthase small chain |
|  | SAKOR_01138 | + | 797/1206 | 266/401 | missense_variant c.797G>A p.Ser266Asn |  | Phosphopantothenoylcysteine decarboxylase |
|  | SAKOR_01265 | + | 885/1062 | 295/353 | synonymous_variant c.885A>T p.Ala295Ala |  | Threonine synthase |
|  | SAKOR_01332 | + | 163/723 | 55/240 | missense_variant c.163G>T p.Ala55Ser |  | Dihydrodipicolinate reductase |
|  | SAKOR_01350 | - | 888/2799 | 296/932 | synonymous_variant c.888A>T p.Ile296Ile |  | 2-oxoglutarate dehydrogenase E1 component |
|  | SAKOR_01367 | - | 7/252 | 3/83 | missense_variant c.7A>T p.Ile3Phe |  | PBS lyase HEAT-like repeat protein |
|  | SAKOR_01460 | - | 18/450 | 6/149 | missense_variant c.18C>A p.Asp6Glu |  | Hypothetical protein |
|  | SAKOR_01604 | - | 369/2631 | 123/876 | synonymous_variant c.369G>A p.Ala123Ala |  | Valyl-tRNA synthetase |
|  | SAKOR_01625 | - | 512/1401 | 171/466 | missense_variant c.512C>A p.Ser171Tyr |  | Replication initiation and membrane attachment protein |
|  | SAKOR_01899 | + | 561/1113 | 187/370 | synonymous_variant c.561G>A p.Arg187Arg |  | Choloylglycine hydrolase |
|  | SAKOR_02022 | + | 641/1047 | 214/348 | missense_variant c.641T>C p.Leu214Ser |  | 3-isopropylmalate dehydrogenase |
|  | SAKOR_02113 | + | 292/321 | 98/106 | missense_variant c.292G>A p.Ala98Thr | *arsR* | Transcriptional regulator, ArsR family protein |
|  | SAKOR_02137 | - | 1183/1188 | 395/395 | missense_variant c.1183A>T p.Ile395Phe |  | UDP-N-acetylglucosamine pyrophosphorylase |
|  | SAKOR_02140 | - | 173/1371 | 58/456 | missense_variant c.173G>T p.Gly58Val |  | Putative membrane spanning protein |
|  | SAKOR_02157 | - | 1101/1413 | 367/470 | synonymous_variant c.1101C>T p.Asn367Asn |  | 6-phospho-beta-galactosidase |
|  | SAKOR_02292 | - | 1363/1605 | 455/534 | missense_variant c.1363T>C p.Tyr455His |  | PTS system, maltose and glucose-specific IIBC component |
|  | SAKOR_02323 | + | 178/420 | 60/139 | missense_variant c.178G>T p.Asp60Tyr |  | hypothetical protein |
|  | SAKOR_02341 | - | 846/1536 | 282/511 | synonymous_variant c.846T>A p.Gly282Gly |  | Malate:quinone oxidoreductase |
|  | SAKOR_02489 | - | 3002/3108 | 1001/1035 | missense_variant c.3002A>G p.Glu1001Gly | *fnbA* | Fibronectin-binding protein fnbA |
|  | SAKOR_02538 | + | 581/2106 | 194/701 | missense_variant c.581T>C p.Leu194Pro |  | hypothetical protein |
|  | SAKOR_02551 | - | 893/1158 | 298/385 | missense_variant c.893A>T p.Asp298Val |  | Aspartate aminotransferase |
|  | SAKOR_02558 | - | 169/786 | 57/261 | stop_gained c.169C>T p.Gln57* | *ssaA* | Secretory antigen precursor SsaA |
|  | SAKOR_02669 | + | 442/549 | 148/182 | missense_variant c.442C>T p.Pro148Ser | *icaB* | N-acetylglucosaminyltransferase, accessory part icaB |
| B | SAKOR_00209 | - | 319/1593 | 107/530 | missense_variant c.319G>A p.Val107Ile |  | Long-chain-fatty-acid--CoA ligase |
|  | SAKOR_01884 | + | 1005/1380 | 335/459 | missense_variant c.1005A>T p.Glu335Asp |  | Aldehyde dehydrogenase (NAD(P)+) |
|  | SAKOR_02091 | - | 650/1260 | 217/419 | missense_variant c.650A>C p.Lys217Thr |  | UDP-N-acetylglucosamine 1-carboxyvinyltransferase |
|  | SAKOR_02616 | - | 1180/1881 | 394/626 | missense_variant c.1180C>T p.Pro394Ser |  | Sulfite reductase [nadph] flavoprotein alpha-component |
| D | SAKOR_00001 | + | 1295/1362 | 432/453 | missense_variant c.1295A>G p.Asp432Gly | *dnaA* | Chromosomal replication initiator protein DnaA |
|  | SAKOR_00020 | + | 968/1362 | 323/453 | missense_variant c.968G>A p.Arg323Lys | *yycH* | Putative membrane protein YycH |
|  | SAKOR_00023 | + | 912/2361 | 304/786 | missense_variant c.912A>T p.Lys304Asn | *sasH* | 5'-nucleotidase |
|  | SAKOR_00105 | + | 518/693 | 173/230 | missense_variant c.518G>A p.Cys173Tyr |  | Undecaprenyl-phosphate galactosephosphotransferase |
|  | SAKOR_00116 | - | 694/816 | 232/271 | missense_variant c.694C>T p.Pro232Ser | *phnE* | Phosphotes transport system permease protein PhnE |
|  | SAKOR_00116 | - | 65/816 | 22/271 | missense_variant c.65G>T p.Arg22Ile | *phnE* | Phosphotes transport system permease protein PhnE |
|  | SAKOR_00132 | + | 1351/1917 | 451/638 | missense_variant c.1351G>A p.Ala451Thr | *capD* | capsular polysaccharide synthesis enzyme CapD |
|  | SAKOR_00134 | + | 441/1116 | 147/371 | synonymous_variant c.441G>T p.Val147Val |  | UDP-2-acetamido-2,6-dideoxy-beta-L-talose 4-dehydrogenase |
|  | SAKOR_00137 | + | 665/1110 | 222/369 | missense_variant c.665C>T p.Pro222Leu | *capI* | Glycosyltransferase |
|  | SAKOR_00172 | + | 308/1455 | 103/484 | missense_variant c.308C>T p.Ser103Leu |  | PTS system, sucrose-specific IIBC component |
|  | SAKOR_00187 | + | 47/1098 | 16/365 | missense_variant c.47A>C p.Asn16Thr |  | hypothetical protein |
|  | SAKOR_00192 | + | 555/1041 | 185/346 | synonymous_variant c.555C>T p.Asp185Asp |  | NAD-dependent oxidoreductase |
|  | SAKOR_00200 | + | 716/756 | 239/251 | missense_variant c.716G>A p.Arg239His |  | Pyruvate formate-lyase activating enzyme |
|  | SAKOR_00224 | + | 916/1260 | 306/419 | missense_variant c.916G>A p.Ala306Thr |  | PTS system, galactitol-specific IIC component |
|  | SAKOR_00406 | + | 874/1086 | 292/361 | missense_variant c.874G>A p.Glu292Lys | *set* | Exotoxin |
|  | SAKOR_00444 | + | 600/1215 | 200/404 | synonymous_variant c.600A>C p.Thr200Thr |  | Cystathionine beta-lyase |
|  | SAKOR_00457 | + | 682/1437 | 228/478 | missense_variant c.682T>G p.Leu228Val |  | PTS system, trehalose-specific IIBC component |
|  | SAKOR_00476 | + | 206/537 | 69/178 | missense_variant c.206C>T p.Thr69Ile |  | Ribonuclease M5 |
|  | SAKOR_00494 | + | 264/1296 | 88/431 | synonymous_variant c.264C>T p.Asn88Asn | *tilS* | TR(Ile)-lysidine synthetase TilS |
|  | SAKOR_00556 | - | 315/702 | 105/233 | missense_variant c.315A>C p.Glu105Asp |  | GlcNAc-PI de-N-acetylase family protein |
|  | SAKOR_00562 | + | 1304/1413 | 435/470 | missense_variant c.1304G>A p.Gly435Glu |  | Proline/betaine transporter |
|  | SAKOR_00624 | + | 508/2043 | 170/680 | missense_variant c.508G>T p.Val170Phe | *nhaP* | Na+/H+ antiporter NhaP |
|  | SAKOR_00651 | + | 360/429 | 120/142 | synonymous_variant c.360G>A p.Gln120Gln |  | hypothetical protein |
|  | SAKOR_00652 | - | 383/507 | 128/168 | missense_variant c.383A>C p.Asn128Thr |  | Acetyltransferase, GT family |
|  | SAKOR_00660 | - | 689/798 | 230/265 | missense_variant c.689G>A p.Gly230Asp | *ssaA* | Secretory antigen precursor SsaA |
|  | SAKOR_00681 | + | 1029/1632 | 343/543 | missense_variant c.1029A>T p.Leu343Phe | *cydD* | Transport ATP-binding protein CydD |
|  | SAKOR_00722 | + | 219/1155 | 73/384 | synonymous_variant c.219A>G p.Gly73Gly |  | Histidinol-phosphate aminotransferase |
|  | SAKOR_00734 | + | 36/171 | 12/56 | synonymous_variant c.36C>T p.Ser12Ser |  | hypothetical protein |
|  | SAKOR_00780 | + | 145/1305 | 49/434 | missense_variant c.145G>A p.Val49Ile |  | Enolase |
|  | SAKOR_00784 | + | 1143/2373 | 381/790 | synonymous_variant c.1143T>A p.Ile381Ile |  | Exoribonuclease II |
|  | SAKOR_00784 | + | 2027/2373 | 676/790 | missense_variant c.2027C>T p.Ala676Val |  | Exoribonuclease II |
|  | SAKOR_00814 | + | 40/894 | 14/297 | missense_variant c.40C>T p.His14Tyr |  | Putative cytosolic protein |
|  | SAKOR_00916 | + | 28/987 | 10/328 | missense_variant c.28G>A p.Glu10Lys |  | Putative competence protein/transcription factor |
|  | SAKOR_00926 | + | 1398/1845 | 466/614 | synonymous_variant c.1398C>T p.Asp466Asp | *napA* | Na+/H+ antiporter NapA |
|  | SAKOR_00929 | + | 1060/1629 | 354/542 | missense_variant c.1060C>T p.His354Tyr |  | Sodium/proton-dependent alanine carrier protein |
|  | SAKOR_00942 | + | 15/1368 | 5/455 | synonymous_variant c.15A>T p.Ser5Ser | *ktrB* | Potassium uptake protein KtrB |
|  | SAKOR_00989 | + | 184/264 | 62/87 | synonymous_variant c.184C>T p.Leu62Leu | *purS* | Phosphoribosylformylglycimidine synthase, PurS component |
|  | SAKOR_01001 | + | 1246/1305 | 416/434 | missense_variant c.1246C>G p.Leu416Val |  | Putative membrane spanning protein |
|  | SAKOR_01016 | + | 742/978 | 248/325 | missense_variant c.742G>A p.Val248Ile |  | Pyruvate dehydrogenase E1 component beta subunit |
|  | SAKOR_01051 | - | 481/1053 | 161/350 | missense_variant c.481G>C p.Val161Leu | *isdA* | Heme uptake heme-iron binding protein IsdA |
|  | SAKOR_01104 | + | 283/432 | 95/143 | missense_variant c.283A>G p.Lys95Glu | *mraZ* | Cell division protein MraZ |
|  | SAKOR_01105 | + | 578/936 | 193/311 | missense_variant c.578A>G p.Lys193Arg |  | 16S rRNA (cytosine(1402)-N(4))-methyltransferase |
|  | SAKOR_01113 | + | 529/792 | 177/263 | missense_variant c.529A>T p.Ser177Cys |  | Putative cytosolic protein |
|  | SAKOR_01154 | + | 293/2061 | 98/686 | missense_variant c.293G>T p.Cys98Phe | *recG* | ATP-dependent D helicase RecG |
|  | SAKOR_01174 | + | 22/1167 | 8/388 | missense_variant c.22A>G p.Ile8Val |  | N-acetylmuramoyl-L-alanine amidase |
|  | SAKOR_01200 | + | 793/972 | 265/323 | missense_variant c.793T>A p.Phe265Ile |  | Riboflavin kinase |
|  | SAKOR_01220 | + | 1042/1545 | 348/514 | missense_variant c.1042G>A p.Glu348Lys |  | TR 2-methylthioadenosine synthase |
|  | SAKOR_01233 | + | 1007/1248 | 336/415 | missense_variant c.1007C>T p.Ala336Val | *hflX* | GTP-binding protein HflX |
|  | SAKOR_01270 | + | 513/1524 | 171/507 | synonymous_variant c.513T>C p.Gly171Gly |  | Catalase |
|  | SAKOR_01287 | + | 1878/2706 | 626/901 | synonymous_variant c.1878C>T p.Asn626Asn |  | Aconitate hydratase |
|  | SAKOR_01304 | + | 932/1476 | 311/491 | missense_variant c.932C>T p.Pro311Leu |  | Anthranilate synthase component I |
|  | SAKOR_01341 | + | 44/279 | 15/92 | missense_variant c.44T>C p.Val15Ala |  | Acylphosphatase |
|  | SAKOR_01365 | - | 88/453 | 30/150 | missense_variant c.88A>G p.Thr30Ala |  | Hypothetical protein |
|  | SAKOR_01373 | - | 12975/13971 | 4325/4656 | synonymous_variant c.12975C>T p.Arg4325Arg |  | Extracellular matrix binding protein |
|  | SAKOR_01373 | - | 12974/13971 | 4325/4656 | missense_variant c.12974G>A p.Arg4325His |  | Extracellular matrix binding protein |
|  | SAKOR_01373 | - | 11185/13971 | 3729/4656 | synonymous_variant c.11185C>T p.Leu3729Leu |  | Extracellular matrix binding protein |
|  | SAKOR_01385 | - | 118/333 | 40/110 | missense_variant c.118A>T p.Asn40Tyr |  | Hypothetical protein |
|  | SAKOR_01393 | - | 61/342 | 21/113 | missense_variant c.61A>C p.Lys21Gln |  | Hypothetical protein |
|  | SAKOR_01507 | - | 333/678 | 111/225 | missense_variant c.333T>G p.Ser111Arg |  | Putative cytosolic protein |
|  | SAKOR_01560 | - | 778/930 | 260/309 | missense_variant c.778A>G p.Thr260Ala |  | Peptidase family U32 |
|  | SAKOR_01574 | - | 93/423 | 31/140 | synonymous_variant c.93T>A p.Ile31Ile | *iscR* | Transcriptional regulator IscR |
|  | SAKOR_01587 | - | 514/1065 | 172/354 | missense_variant c.514G>C p.Asp172His |  | S-adenosylmethionine:tRNA ribosyltransferase-isomerase |
|  | SAKOR_01604 | - | 2095/2631 | 699/876 | missense_variant c.2095G>T p.Gly699Cys |  | Valyl-tRNA synthetase |
|  | SAKOR_01605 | + | 111/600 | 37/199 | synonymous_variant c.111G>A p.Pro37Pro |  | DNA-3-methyladenine glycosylase |
|  | SAKOR_01620 | - | 186/603 | 62/200 | missense_variant c.186A>T p.Glu62Asp |  | Bacterial Protein Translation Initiation Factor 3 (IF-3) |
|  | SAKOR_01689 | - | 40/885 | 14/294 | missense_variant c.40G>A p.Val14Ile |  | Metal-dependent hydrolase |
|  | SAKOR_01697 | + | 821/1284 | 274/427 | missense_variant c.821C>T p.Ala274Val |  | NAD(FAD)-utilizing dehydrogenase |
|  | SAKOR_01697 | + | 892/1284 | 298/427 | missense_variant c.892C>G p.Pro298Ala |  | NAD(FAD)-utilizing dehydrogenase |
|  | SAKOR_01789 | - | 363/1236 | 121/411 | synonymous_variant c.363T>A p.Ile121Ile | *ecsB* | Protein EcsB |
|  | SAKOR_01796 | + | 912/963 | 304/320 | missense_variant c.912A>T p.Glu304Asp |  | Peptidyl-prolyl cis-trans isomerase |
|  | SAKOR_01799 | - | 603/1197 | 201/398 | missense_variant c.603A>T p.Gln201His |  | Phosphoesterase |
|  | SAKOR_01899 | + | 279/1113 | 93/370 | missense_variant c.279G>A p.Met93Ile |  | Choloylglycine hydrolase |
|  | SAKOR_02036 | - | 328/360 | 110/119 | missense_variant c.328A>G p.Ser110Gly |  | Holo-[acyl-carrier protein] synthase |
|  | SAKOR_02104 | - | 538/1341 | 180/446 | synonymous_variant c.538T>C p.Leu180Leu |  | Thymidine phosphorylase |
|  | SAKOR_02123 | + | 1509/1539 | 503/512 | synonymous_variant c.1509A>G p.Leu503Leu |  | PTS system, mannitol-specific IIBC component |
|  | SAKOR_02136 | - | 543/693 | 181/230 | synonymous_variant c.543C>T p.Phe181Phe |  | Conserved membrane protein (hemolysin III -like protein) |
|  | SAKOR_02144 | - | 525/1014 | 175/337 | synonymous_variant c.525T>C p.Asn175Asn |  | Iron(III) dicitrate-binding protein |
|  | SAKOR_02149 | + | 1849/1977 | 617/658 | missense_variant c.1849G>A p.Glu617Lys | *sfaD* | Siderophore synthase |
|  | SAKOR_02162 | - | 441/516 | 147/171 | missense_variant c.441C>A p.Ser147Arg | *lacB* | Galactose-6-phosphate isomerase lacB subunit |
|  | SAKOR_02171 | + | 89/168 | 30/55 | missense_variant c.89C>T p.Thr30Met |  | hypothetical protein |
|  | SAKOR_02219 | + | 549/864 | 183/287 | synonymous_variant c.549A>G p.Ala183Ala |  | Glucose uptake protein |
|  | SAKOR_02276 | - | 2127/2964 | 709/987 | synonymous_variant c.2127A>C p.Thr709Thr |  | Formate dehydrogenase alpha chain |
|  | SAKOR_02290 | - | 365/960 | 122/319 | missense_variant c.365G>A p.Gly122Asp |  | Transporter, Sodium/bile acid symporter family protein |
|  | SAKOR_02347 | + | 631/1047 | 211/348 | missense_variant c.631G>A p.Ala211Thr |  | Alcohol dehydrogenase |
|  | SAKOR_02389 | + | 248/1251 | 83/416 | stop_gained c.248C>G p.Ser83* |  | UDP-N-acetylmuramoylheptapeptide-glycine L-seryltransferase |
|  | SAKOR_02421 | - | 43/828 | 15/275 | missense_variant c.43G>A p.Asp15Asn |  | Phosphoesterase |
|  | SAKOR_02436 | - | 675/1209 | 225/402 | synonymous_variant c.675G>A p.Thr225Thr |  | Chloramphenicol resistance protein |
|  | SAKOR_02479 | + | 318/1836 | 106/611 | synonymous_variant c.318G>A p.Ala106Ala |  | Phosphoglucomutase |
|  | SAKOR_02484 | - | 5080/5274 | 1694/1757 | missense_variant c.5080G>A p.Glu1694Lys |  | Beta-N-acetylhexosaminidase |
|  | SAKOR_02496 | - | 45/489 | 15/162 | missense_variant c.45G>T p.Met15Ile |  | Hypothetical protein |
|  | SAKOR_02518 | + | 190/492 | 64/163 | missense_variant c.190T>G p.Phe64Val |  | Acetyltransferase, GNAT family protein |
|  | SAKOR_02536 | + | 361/1167 | 121/388 | missense_variant c.361G>A p.Ala121Thr |  | Hydroxymethylglutaryl-CoA synthase |
|  | SAKOR_02540 | - | 1466/2031 | 489/676 | stop_gained c.1466T>A p.Leu489* |  | Ferrous iron transport protein B |
|  | SAKOR_02548 | + | 510/2409 | 170/802 | synonymous_variant c.510A>G p.Pro170Pro |  | Copper-exporting ATPase |
|  | SAKOR_02572 | + | 525/558 | 175/185 | synonymous_variant c.525C>T p.Asn175Asn | *tetR* | Transcriptional regulator, TetR family protein |
|  | SAKOR_02602 | - | 1208/1620 | 403/539 | missense_variant c.1208A>T p.Tyr403Phe |  | Acetyl-coenzyme A synthetase |
|  | SAKOR_02638 | - | 142/1530 | 48/509 | missense_variant c.142G>T p.Asp48Tyr | *aur* | Zinc metalloproteise aureolysin |
|  | SAKOR_02693 | + | 943/957 | 315/318 | missense_variant c.943C>T p.His315Tyr |  | Rhodanese-related sulfurtransferase |

^a^Nucleotide position of the variant within the ORFs; ^b^Amino acid position of the variant within the ORFs.
